# E3 Ubiquitin Ligase Nedd4‐2 Exacerbates Seizure‐Induced Mitochondrial Defects in an Alzheimer's Disease Mouse Model

**DOI:** 10.1111/jnc.70440

**Published:** 2026-04-14

**Authors:** Yingxin Wang, Jiuhe Zhu, Simon Lizarazo, Kwan Young Lee, Olivia Wong, Sophia Azim, Yeeun Yook, Vipendra Kumar, Nien‐Pei Tsai

**Affiliations:** ^1^ Department of Molecular and Integrative Physiology School of Molecular and Cellular Biology, University of Illinois at Urbana‐Champaign Urbana Illinois USA; ^2^ Neuroscience Program, University of Illinois at Urbana‐Champaign Urbana Illinois USA; ^3^ Beckman Institute, University of Illinois at Urbana‐Champaign Urbana Illinois USA; ^4^ Cancer Center at Illinois, University of Illinois at Urbana‐Champaign Urbana Illinois USA

**Keywords:** Alzheimer's disease, mitochondria, mitofusion 2, Nedd4‐2, seizures, ubiquitination

## Abstract

Seizure is one of the common comorbidities in Alzheimer's disease (AD). Seizures in AD have been shown to occur more often with early‐onset disease, particularly when there is a familial presenilin I (PS1) mutation or abnormal expression of amyloid precursor protein (APP). AD patients with seizures have been associated with a faster decline in cognitive functions. However, it remains unclear how seizures exacerbate neurodegeneration in AD. Here, we showed that, using a kainic acid‐induced acute seizure model, mitochondrial function is enhanced and the reactive oxygen species (ROS) are reduced in the brain of wild‐type (WT) mice but not in an AD mouse model, APP/PS1 mice. These data suggest a lack of protective mechanism following seizures in APP/PS1 mice. Mechanistically, we found that an E3 ubiquitin ligase, the neural precursor cell‐expressed developmentally downregulated protein 4‐like (Nedd4‐2), is elevated but stays dephosphorylated in APP/PS1 mice upon seizure inductions. Immunocytochemistry and sub‐cellular fractionation experiments demonstrate an interaction between Nedd4‐2 and mitochondria. Unbiased proteomics analysis suggests that Nedd4‐2 regulates the expression of multiple mitochondrial proteins including one of the key mitochondrial outer membrane proteins, Mitofusin 2 (MFN2). Upon seizure induction, Nedd4‐2 exhibits elevated interaction with mitochondria and downregulates MFN2 in APP/PS1 mice but not in WT mice. These data suggest that seizures aggravate mitochondrial dysfunction in AD, and Nedd4‐2, which acts as a negative mitochondrial regulator, contributes to this effect. Altogether, our findings illustrate a potential mechanism by which seizures exacerbate neurodegeneration in AD and suggest Nedd4‐2 as a novel therapeutic target for AD patients with comorbid seizures.

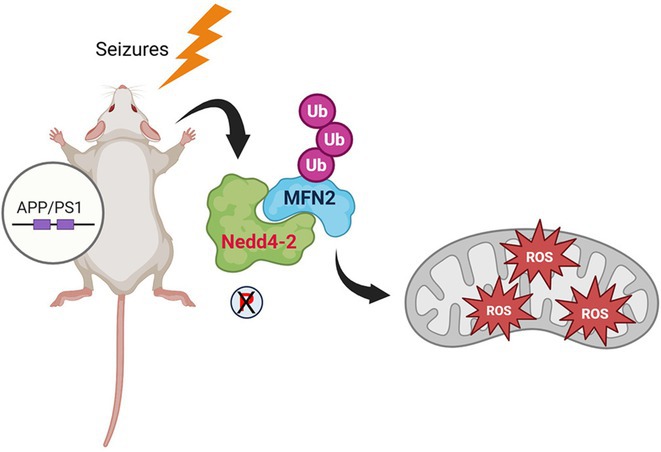

## Graphical abstract

Seizures are a common comorbidity in Alzheimer's disease (AD), especially in early‐onset cases linked to PS1 mutations or abnormal APP expression, and are associated with accelerated cognitive decline. However, the mechanisms by which seizures worsen AD pathology remain unclear. This study shows that acute seizures enhance mitochondrial function and reduce reactive oxygen species in wild‐type mice, but not in APP/PS1 AD mice, indicating a loss of protective responses. Mechanistically, seizures alter the activity of the E3 ubiquitin ligase Nedd4‐2 in APP/PS1 mice, leading to mitochondrial dysfunction via downregulation of Mitofusin 2. These findings suggest that Nedd4‐2–mediated mitochondrial dysregulation contributes to seizure‐exacerbated neurodegeneration in AD and represents a potential therapeutic target.

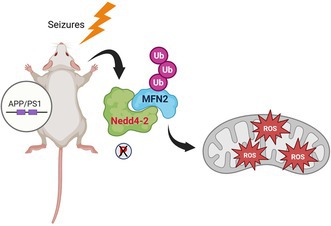

**Abbreviations**
AD: Alzheimer's disease
APP: amyloid precursor protein
Aβ: amyloid‐β
cKO: conditional knockout
COX IV: cytochrome C oxidase subunit 4
DIV: days‐in vitro
DMSO: Dimethyl sulfoxide
GAPDH: glyceraldehyde‐3‐phosphate dehydrogenase
HEK: human embryonic kidney
MAP 2: microtubule‐associated protein 2
MFN2: mitofusin 2
Nedd4‐2: neural precursor cell‐expressed developmentally downregulated protein 4‐like
NRF1: nuclear respiratory factor 1
PS1: presenilin I
ROS: reactive oxygen species
TOM20: translocase of outer mitochondrial membrane 20
VDAC: voltage‐dependent anion channel
WT: wild‐type

## 1 Introduction

Reduced neuronal excitability and synaptic transmission are thought to contribute to cognitive defects in AD (Zhang et al. 2021). However, it is increasingly recognized that many patients with AD in fact experience brain hyperexcitability and recurrent seizures, especially starting during the early phase of AD progression (30–50 years old in patients and 8–16 weeks in mouse models) (Ghatak et al. 2019; Ping et al. 2015). The seizure associated with AD is particularly apparent in patients who carry a familial presenilin I (PS1) mutation or have elevated expression of amyloid precursor protein (APP) (Vossel et al. 2013, 2017). As observed previously, AD patients with seizures are associated with a worsened disease trajectory (Vossel et al. 2016, 2017), producing significant burden to the patients as well as the caregivers. Although our previous work has revealed the mechanism underlying higher seizure susceptibility during the early stage of AD (Yook et al. 2024), it remains unclear how seizures exacerbate neurodegeneration in AD.

A substantial number of clinical studies have observed defects in mitochondria in AD patients as well as postmortem patient samples (Terada et al. 2020; Troutwine et al. 2022). This is consistent with altered energy metabolism and elevated oxidative stress frequently observed in patients and animal models of AD (Yao et al. 2009; Yoo et al. 2020). Mitochondria dysfunction in AD has been linked to the toxicity of intracellular amyloid‐β (Aβ) peptides (Reddy and Beal 2008). Several molecular mechanisms underlying Aβ's impact on mitochondria have been proposed. For example, Aβ can be localized to mitochondria and disrupt the electron transport chain (Manczak et al. 2006). In addition, Aβ binds to alcohol dehydrogenase and facilitates the production of reactive oxygen species (ROS) (Lustbader et al. 2004). Indirectly, Aβ‐induced hyperexcitability also causes an elevation of intracellular calcium, leading to enhanced mitochondrial membrane depolarization and impaired energy production (Calvo‐Rodriguez et al. 2020). Despite these past discoveries, however, there is still a lack of approaches for addressing the mitochondrial damage caused by Aβ other than directly antagonizing Aβ. Given the controversial and ambiguous findings about the efficacy of anti‐Aβ antibody in clinical studies, there remains a need to identify new druggable pathways to improve mitochondrial health in AD.

To address the questions surrounding comorbid seizures and mitochondrial defects in AD, we studied the neural precursor cell‐expressed developmentally downregulated gene 4‐like (*Nedd4‐2*, also known as *Nedd4L*). *Nedd4‐2* encodes a HECT (homologous to the E6‐AP C‐terminus)‐type E3 ubiquitin ligase that is highly expressed in the brain. Because of a C‐terminal lipid binding domain, Nedd4‐2 has a high affinity toward binding and ubiquitinating membrane‐bound proteins (Zhu et al. 2019). Insufficient function of Nedd4‐2 has been linked to multiple neurological and psychiatric disorders, including epilepsy, autism spectrum disorders, and periventricular nodular heterotopia (Broix et al. 2016; Lee et al. 2021; Zhu et al. 2017). With regard to neurodegeneration, a previous study has shown that Nedd4‐2 is indirectly involved in altered autophagy signaling (Zhang et al. 2020). However, it remains unclear whether Nedd4‐2 is dysregulated in AD, and if so, what role it may play.

In this study, we first observed that chemically induced seizures elevate the expression of a critical mitochondria gene, nuclear respiratory factor 1 (NRF1) and reduce reactive oxygen species (ROS) in the brain of WT mice but not APP/PS1 mice, a well‐established mouse model of familial AD. Because hyperexcitability can cause mitochondrial stress (Andreasen and Nedergaard 2021; Norat et al. 2020), we suspect that acute seizures trigger a protective mechanism to mitigate ROS, but such a mechanism is impaired in APP/PS1 mice. To determine the underlying mechanism, we found that Nedd4‐2 is elevated but stays dephosphorylated in APP/PS1 mice but not in WT mice following seizure induction. We further showed that dephosphorylated Nedd4‐2 exerts a previously unknown function toward ubiquitinating one of the key mitochondrial outer membrane proteins, Mitofusin 2 (MFN2). Upon seizure induction, Nedd4‐2 exhibits an elevated interaction with mitochondria and downregulates MFN2 in APP/PS1 mice but not in WT mice. Together, our data suggest that APP/PS1 mice are prone to seizure‐induced mitochondrial dysfunction, and Nedd4‐2 potentially contributes to this defect. Our results may suggest Nedd4‐2 as a novel therapeutic target for mitigating neurodegeneration with comorbid seizures in AD.

## 2 Materials and Methods

This study followed the guidelines of Animal Care and Use provided by the University of Illinois Institutional Animal Care and Use Committee (IACUC) and the guidelines of Euthanasia of Animals provided by the American Veterinary Medical Association (AVMA) to minimize the number of animals used and animal suffering. This study was conducted under an approved IACUC animal protocol at the University of Illinois at Urbana‐Champaign (23016 to N.‐P.T).

### 2.1 Mice

Wild‐type (WT) (JAX cat. no. 000664), *Emx1*‐Cre mice (JAX cat. no. 022762), and APP/PS1 double transgenic mice (JAX cat. no. 005864, also MMRRC_034832‐JAX) were backcrossed in a C57BL/6J background. *Nedd4–2* floxed mice were generated and obtained from Dr. Hiroshi Kawabe (Max Planck Institute) (Kimura et al. 2011). Up to five mice per cage were housed in individually ventilated cages in a 12 h/12 h light/dark cycle in a temperature‐controlled room with *ad libitum* access to water and food. For making primary neuronal cultures, mice at postnatal (P) day 0–1 were anesthetized on ice for 2 min before immediate decapitation. For collecting tissues, young adult mice were anesthetized by 1% isoflurane inhalation for 2 min. After confirming the loss of movement and tail pinch reflex, decapitation was then performed. For genotyping, the primers to detect WT and mutant alleles for APP/PS1 were as follows: 5′‐GTG TGA TCC ATT CCA TCA GC‐3′ (WT forward), 5′‐GGA TCT CTG AGG GGT CCA GT‐3′ (Common reverse), and 5′‐ATG GTA GAG TAA GCG AGA ACA CG‐3′ (Mutant forward). The primers to detect the *Nedd4–2 loxP* allele are as follows: 5′‐TCCCCACTGCAGTTCCTACC‐3′, and 5′‐AGCTGCTCAGGCTGAATCACC‐3′. The primers used to detect the *Emx1* allele are 5′‐CGGTCTGGCAGTAAAAACTATC‐3′ (*Emx1‐Cre*), 5′‐GTGAAACAGCATTGCTGTCACTT‐3′ (*Emx1‐Cre*); 5′‐AAGGTGTGGTTCCAGAATCG‐3′ (*Wild type*), and 5′‐CTCTCCACCAGAAGGCTGAG‐3′ (*Wild type*). Polymerase chain reactions (PCR) were performed using the setting as below: 2 min at 94°C, 10 cycles of 20 s at 94°C, 15 s at 65°C (decreased by 0.5°C per cycle), and 10 s at 68°C; 28 cycles of 15 s at 94°C, 15 s at 60°C, and 10 s at 72°C; and 2 min at 72°C.

### 2.2 Reagents

Kainic acid was from Hellobio (cat. no. HB0355) and saline was from Hanna Pharmaceutical Supply. Puromycin was from Cayman Chemical (cat. no. 13884). The antibodies used in this study are from GenScript Corporation (anti‐Gapdh [RRID:AB_2107436]), Cell Signaling (anti‐Nedd4‐2 [RRID:AB_1904063], anti‐NRF1 [RRID:AB_2732888], anti‐COX‐IV [RRID:AB_2797784], anti‐TOM20 [RRID:AB_2687663], anti‐VDAC [RRID:AB_10557420], anti‐Myc [RRID:AB_490778], anti‐β‐actin [RRID:AB_2242334], anti‐ubiquitin [RRID:AB_3075532], anti‐NeuN [RRID:AB_2651140], and anti‐mouse IgG [RRID:AB_330924]), ProteinTech (anti‐MFN2 [RRID:AB_2882713]), Jackson Immuno Research (anti‐rabbit IgG, [RRID:AB_10015282]), Millipore (anti‐puromycin, [RRID:AB_2566826]), Abcam (anti‐MAP2, RRID:AB_2138147) and ThermoFisher (Goat anti‐Rabbit IgG (H+L) Alexa Fluor 555 [RRID:AB_141784]. Goat anti‐Chicken IgY (H+L) Alexa Fluor 633 [RRID:AB_2535756] and Goat anti‐Mouse IgG (H+L) Alexa Fluor 488 [RRID:AB_2534069]).

### 2.3 Immunoprecipitation and Western Blotting

For immunoprecipitation (IP), cell lysates were obtained by sonicating pelleted cells in a IP buffer (50 mM Tris, pH 7.4, 120 mM NaCl, 0.5% Nonidet P‐40). Eighty μg of total protein lysates, measured using the Bradford assay, were incubated for 2 h at 4°C with 0.5 μg primary antibody. Protein A/G agarose beads (Santa Cruz Biotechnology cat. no. sc‐2003) were added for another hour followed by two 10‐min washings with IP buffer. Protein samples for western blotting were mixed in a sodium dodecyl sulfate (SDS) buffer (40% glycerol; 240 mM Tris–HCl, pH 6.8; 8% sodium dodecyl sulfate; 0.04% bromophenol blue; and 5% β‐mercaptoethanol) and boiled for 5 min. After cooling on ice for 3 min, the samples were loaded onto either 8%, 10%, or 12% sodium dodecyl sulfate polyacrylamide gel electrophoresis gels. After gel electrophoresis, the gel was transferred onto a polyvinylidene fluoride membrane. The membrane was blocked with 1% bovine serum album solution in Tris‐buffered saline Tween‐20 buffer (TBST; [20 mM Tris, pH 7.5; 150 mM NaCl; 0.1% Tween‐20]) and then incubated overnight with primary antibody at 4°C. On the following day, the membranes were washed with TBST three times for 10 min each and then incubated with secondary antibodies in 5% milk in TBST for 1 h at room temperature (24°C). After washing the membranes with TBST for 10 min three times, the image was developed using iBright FL 1500 (Invitrogen). For image analysis, ImageJ software was used to quantify the intensity of protein bands. Normalization was done by normalizing the intensity of protein of interest to GAPDH or β‐actin (total lysates) or COX IV (mitochondrial fractions). Uncropped full images of Western blotting results are provided in Appendix S1.

### 2.4 Seizure Induction

Mice at 10 weeks of age were intraperitoneally injected with kainic acid, prepared in saline solution at doses of 15 mg/kg. The total injection volume was kept close to 0.15 mL. After injection, mice were closely observed in real time for 2 h, immediately followed by brain harvesting and sample processing.

### 2.5 Cell Cultures

Cortices from 3 to 4 mice were collected, pooled, digested with trypsin and triturated in Dulbecco's Modified Eagle Medium (DMEM) with DNase‐I (1 mg/mL). Two to three hours after cells were placed on Poly‐D‐Lysin (PDL)‐coated plates, the DMEM was replaced with NeuralA basal medium supplemented with B27 supplement, 2 mM GlutaMax, a mix of the antibiotics Penicillin (10 000 IU) and Streptomycin (10 000 μg/mL), and 1 μM cytosine β‐D‐arabinofuranoside (AraC). Half of the culture medium was changed with fresh medium every 3–4 days until the cells were harvested at days‐in vitro (DIV) 12–14. Human Embryonic Kidney (HEK) 293 cells, which are not listed as commonly misidentified by the International Cell Line Authentication Committee, were maintained in DMEM with 10% fetal bovine serum (FBS) and split every 3 days. The number of passages for cells is limited to 20. Because these cells are used only to serve as an in vitro platform to measure ubiquitination of exogenously expressed proteins, no authentication was performed. Each experiment in this study was conducted with cultures made from at least three independent litters.

### 2.6 Proteomics

The proteomic screening was conducted using cytosolic fractions of *Nedd4‐2* WT (Nedd4‐2^f/f^ Cre^−^) and *Nedd4‐2* conditional knockout (cKO; Nedd4‐2^f/f^ Cre^+^) mouse brains with label‐free analysis provided by Bioproximity Inc. Following the protein extraction, trypsin was added at a ratio of 1:50 to the samples, which were then incubated at 37°C overnight. Following that, the peptides were extracted, lyophilized, and resuspended in 2–20 μL of 0.1% formic acid. Ultra‐performance liquid chromatography–tandem mass spectrometry (UPLC‐MS/MS) was done using the Easy‐nLC1200 and HF‐X Hybrid Quadropole‐Orbitrap mass spectrometer. The relative protein abundance was determined by the chromatographic peak intensity measurements, done by aligning the chromatographic peaks of precursor ions. The analysis including false discovery rate was done using MaxQuant software (1.6.2.14) and the parameters were set as follows: the protein modifications were carbamidomethylation (C) (fixed), oxidation (M) (variable); the enzyme specificity was set to trypsin; the maximum missed cleavages were set to 2; the precursor ion mass tolerance was set to 10 ppm, and MS/MS tolerance was 20 ppm. Only high confident identified peptides were chosen for downstream protein identification analysis. The relative intensity of each identified protein from the sample sets was normalized to the intensity of β‐actin. The mean and the subsequent standard deviation from the mean, used to create the heat maps, were derived from the pooled average of individual genes from all samples. The raw data files were analyzed and searched against the Uniprot‐ *Mus musculus* protein databases.

### 2.7 Mitochondrial Isolation

Mitochondria were isolated from brains snap‐frozen in liquid nitrogen and stored at −80°C. Mitochondria isolation kit (Thermo Scientific, cat. no. 89801) was employed to perform mitochondrial isolation. In brief, tissues were transferred and homogenized in Dounce homogenizer with 800 mL of phosphate buffered saline (PBS). The tissue suspensions were transferred to 2‐ml microcentrifuge tubes and centrifuged at 1000 × *g* for 3 min at 4°C. Following this, 800 mL of Reagent A with 4 mg/mL BSA and proteinase inhibitor were added to the pellet. Following a brief vortex for 5 s and incubation on ice for 2 min, 10 mL of Mitochondrial Isolation Reagent B was added immediately and vortexed at maximum speed for 5 s every minute for 5 min. The samples were then centrifuged at 700 × *g* for 10 min at 4°C and the supernatant was transferred to a clean 2‐ml tube. The supernatant was then centrifuged again at 3000 × *g* for 15 min at 4°C. The remaining supernatant is cytosolic fractions. We then added 500 mL of Wash Buffer to the pellet and centrifuged at 12 000 × *g* for 5 min. The pellet is purified mitochondrial fractions.

### 2.8 Measurement of Reactive Oxygen Species (ROS)

ROS assay was performed using Amplex Red Hydrogen Peroxide/Peroxidase Assay Kit (Thermo Scientific, cat. no. A221880). Mitochondria used for ROS assay were freshly isolated from forebrain tissues. The mitochondrial pellet was resuspended in 1× Reaction Buffer provided by the kit. H_2_O_2_ standard curve was generated based on the manufacturer's protocol. 50 mL working solution of 100 mM Amplex Red reagent with 0.2 U/mL Horseradish Peroxidase was added to each well containing H_2_O_2_ standard solutions or resuspended mitochondrial samples. The plate was incubated at room temperature (24°C) for 30 min in darkness, followed by measuring the absorbance at 590 nm. The ROS levels in mitochondrial samples were calculated based on H_2_O_2_ standard curve and normalized to the protein concentration of corresponding mitochondrial samples.

### 2.9 Measurement of JC‐1

Primary cortical neurons were cultured on PDL‐coated 96‐well plates. On DIV 14, neurons were washed with PBS and incubated with JC‐1 dye (20 μM) for 10 min at 37°C. After washing with PBS twice, the plates were read by a spectrophotometer. The aggregates were detected with excitation and emission at 535 nm and 590 nm, respectively, while the green monomers were detected with excitation and emission at 485 nm and 535 nm, respectively.

### 2.10 Immunocytochemistry With Dissociated Neurons

Primary cortical neurons on glass coverslips at a density of 1.5 × 10^5^ cells were used. The cells were washed once with PBS, fixed with fixation buffer (4% paraformaldehyde and 4% sucrose in PBS) for 15 min, and then permeabilized with 0.5% Triton‐X‐100 in PBS for 5 min at room temperature. Following fixation and permeabilization, the neurons were incubated overnight with primary antibodies in 1% BSA in PBS. Following the overnight incubation, the samples were washed three times for 10 min each with PBS, incubated with Alexa Fluor conjugated secondary antibodies for 2 h at room temperature in 1% BSA in PBS, and then were washed an additional three times for 10 min each with PBS. The coverslips were then mounted onto glass slides. Images were obtained using a Zeiss LSM 700 confocal microscope with 40× magnification and three different laser lines (488, 555, and 633 nm). The pinhole was set to 1 airy unit.

### 2.11 Immunohistochemistry With Hippocampal Sections

Mice were anesthetized using isoflurane inhalation and transcardially perfused with PBS containing 10 units/mL heparin sodium (ThermoFisher Scientific, cat. no. 411210010) followed by 4% paraformaldehyde (PFA). The brains harvested were stored in 4% PFA overnight and then transferred to 10%, 20%, and 30% sucrose solution every 24 h at 4°C. Brains were then cryosectioned in Leica 3050S cryotome and 25 μm sections were obtained. For immunostaining, sections were placed on gelatin‐coated slides and incubated in antigen unmasking solution (Vector Labs, cat. no. H‐3300) at 70°C for 40 min in a water bath. Sections were washed 3 times with PBS for 5 min each and permeabilized with 0.3% triton‐x 100 for 10 min and then blocked with blocking buffer (1% bovine serum albumin, 3% normal goat serum and 0.3% triton‐x 100) for 1 h at 25°C. Sections were then probed with primary antibody prepared in blocking buffer and incubated overnight at 4°C with primary antibodies. Sections were washed with PBS and probed with Alexa488‐and Alex555‐conjugated secondary antibodies prepared in PBS at 1:500 dilution and incubated for 2 h at 25°C. Sections were washed with PBS and mounted using a mounting medium. Imaging was performed in Zeiss LSM 880 Confocal microscope using 555 and 488 nm lasers. Images were acquired with 60X objective at 1X digital zoom and were processed using ImageJ software (National Institute of Health).

### 2.12 Experimental Design and Statistical Analysis

At least 3 L were used in each experiment; the figure legends provide specific information regarding the sample number for each experiment. No randomization was performed to allocate subjects in this study. No exclusion criteria were pre‐determined and no mice were excluded. Because of the design of our research, no blinding was performed. Power analysis was conducted using G*Power 3.1 to calculate group sizes with power set at 0.8 and α value set at 0.05. The effect size was estimated based on four of our recent studies that employed similar techniques (Kumar et al. 2024; Lee et al. 2023, 2021; Tsai et al. 2017). Outlier was determined by Grubb's outlier test. Statistical methods to determine significance along with sample numbers were indicated in each figure legend. In brief, ANOVA with post hoc Tukey HSD (Honest Significant Differences) test was used for multiple comparisons between treatments or genotypes. Student's *t*‐test was used for analyzing samples with two groups. Tests were performed two‐tailed. Differences are considered significant at the level of *p* < 0.05. No nonparametric analysis was done in our study. The data presented in this study have been tested for normality using the Kolmogorov–Smirnov test and passed the test. Data analyses were performed using GraphPad Prism (Version 10). A total of 82 mice were used for brain tissue collection. Because no sex differences were observed in our results, all data were combined from both male and female mice. Full statistical reports are summarized in Table S1.

## 3 Results

### 3.1 Acute Seizures Potentiate Mitochondrial Functions in WT but Not in APP/PS1 Mice

**FIGURE 1:**
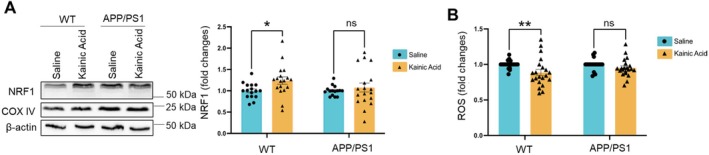
Acute seizures potentiate mitochondrial functions in WT but not in APP/PS1 mice. (A, B) Representative western blots and quantification of nuclear respiratory factor 1 (NRF1) and β‐Actin in total brain lysates (A) as well as quantification of reactive oxygen species in purified mitochondria (B) from 10‐week‐old wild‐type (WT) and APP/PS1 mice intraperitoneally injected with saline or kainic acid (15 mg/kg) for 2 h (*n* = 16–18 mice per group). Two‐way ANOVA with Tukey's test was used. Data are represented as mean ± SEM with *p < 0.05, **p < 0.01, ns: non‐significant.

As a common comorbidity of Alzheimer's disease (AD), seizure can accelerate the progression of AD (Giorgi et al. 2020; Voglein et al. 2020). However, the mechanism remains largely unknown. To begin answering this question, we study mitochondria, the cellular factory that produces energy. Mitochondria are known to play an important role in regulating neuronal excitability (von Ruden et al. 2020). However, how seizure affects mitochondrial function, especially in AD, remains unclear. To address this question, we employed a well‐established seizure model in mice via intraperitoneal injections of kainic acid (15 mg/kg) (Liu et al. 2021; Yook et al. 2024) and measured the expression of several key mitochondrial proteins or regulators, including Translocase of Outer Mitochondrial Membrane 20 (TOM20), Voltage‐Dependent Anion Channel (VDAC), and Nuclear Respiratory Factor 1 (NRF1). Following injections of kainic acid (15 mg/kg) to induce seizures for 2 h in mice, we observed an elevation of NRF1 (Figure 1A), but not TOM20 or VDAC1 (Figure S1), in wild‐type (WT) mice. This observation suggests that the effect of seizures on NRF1 may be selective. Because NRF1 is crucial to mitochondrial biogenesis, we ask whether acute seizures potentiate mitochondrial functions in WT mice. To test this possibility, we measured the levels of reactive oxygen species (ROS) in purified mitochondria from WT mice 2 h after kainic acid injections. As shown in Figure 1B, we observed a reduction of ROS in WT mice. Because previous studies observed elevated ROS in rodent models 24 h after kainic acid–induced seizures (Dabbeni‐Sala et al. 2001; Gilberti and Trombetta 2000), our findings suggest an acute increase in mitochondrial function and a reduction in ROS before the cells enter a state of severe oxidative stress. Most importantly, we did not observe an elevation of NRF1 or a reduction of ROS following kainic acid‐induced seizures in a familial AD mouse model, the APP/PS1 mice (APPswe, PSEN1dE9; JAX 005864). Together, our data suggest that acute seizures increase mitochondrial functions in WT mice potentially through NRF1‐associated mechanisms and such an effect is absent in APP/PS1 mice.

### 3.2 Acute Seizures Differentially Regulate Nedd4‐2 in WT and APP/PS1 Mice

**FIGURE 2:**
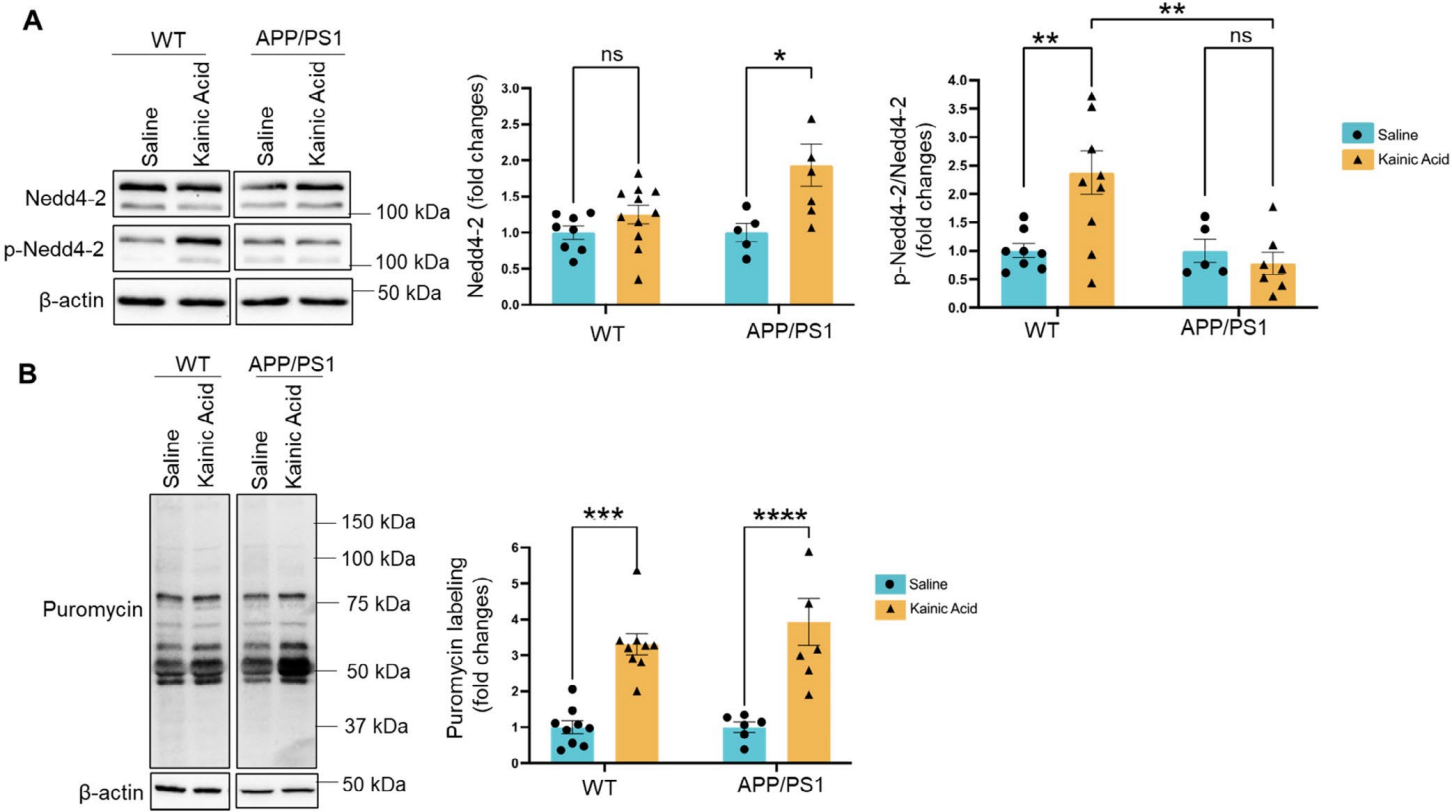
Acute seizures elevate the expression of Nedd4‐2 but keep it dephosphorylated in APP/PS1 mice. (A) Representative western blots and quantification of neural precursor cell‐expressed developmentally downregulated protein 4‐like (Nedd4‐2) and phosphorylated Nedd4‐2 at S342 residue (p‐Nedd4‐2) as well as β‐Actin in total brain lysates from 10‐week‐old WT and APP/PS1 mice intraperitoneally injected with saline or kainic acid (15 mg/kg) for 2 h (*n* = 5–11 mice per group). Both bands on Nedd4‐2 blots were included in data analysis. (B) Representative western blots and quantification of puromycin labeling in total brain lysates from 10‐week‐old WT and APP/PS1 mice intraperitoneally injected with saline or kainic acid (15 mg/kg) together with puromycin (200 mg/kg) for 1 h (*n* = 6–11 mice per group). One outlier in WT mice treated with kainic acid was removed. Two‐way ANOVA with Tukey's test was used. Data are represented as mean ± SEM with *p < 0.05, **p < 0.01, ***p < 0.001, ****p < 0.0001, ns: non‐significant.

Neural precursor cell‐expressed developmentally downregulated gene 4‐like, or Nedd4‐2, is an E3 ubiquitin ligase that is highly expressed in the brain. Nedd4‐2 is encoded by an epilepsy‐associated gene (Allen et al. 2013). Haploinsufficiency of Nedd4‐2 as well as missense mutations of Nedd4‐2 have been linked to elevated susceptibility to epileptic seizures (Zhu et al. 2017, 2019). To explore whether and how Nedd4‐2 affects mitochondrial functions after seizures, WT and APP/PS1 mice were injected with kainic acid (15 mg/kg) for 2 h. We followed by measuring the levels of Nedd4‐2 and the phosphorylation status of Nedd4‐2 at serine‐342 residue (S342) in brain lysates using western blotting. As shown in Figure 2A, Nedd4‐2 level is increased in APP/PS1 mice following kainic acid injections. However, such an elevation did not occur in WT mice. Interestingly, Nedd4‐2 phosphorylation at S342 is elevated in WT mice but not APP/PS1 mice after kainic acid injections. Please note that the two bands in the Nedd4‐2 blots represent the two major isoforms of Nedd4‐2 (Zhu et al. 2019). Therefore, both bands were included in the data analysis. These results indicate that Nedd4‐2 is differentially regulated, in regard to both its expression and phosphorylation, in WT mice versus APP/PS1 mice following seizures.

Because our published work has shown that Nedd4‐2 can interact with ribosomes and repress protein synthesis (Eagleman et al. 2021) while seizures can induce transient protein synthesis (Liu et al. 2019), we ask whether an elevation of Nedd4‐2 reduces protein synthesis in APP/PS1 mice following seizures. To answer this question, we used the surface sensing of translation (SUnSET) technique to measure protein synthesis by labeling newly synthesized protein with puromycin. The mice were intraperitoneally injected with saline or kainic acid (15 mg/kg) together with puromycin (200 mg/kg) for 1 h before the brains were harvested for western blotting with anti‐puromycin antibody. As shown in Figure 2B, both WT and APP/PS1 showed significant elevation of protein synthesis after kainic acid injections. These results indicate that the differential expression of Nedd4‐2 in WT and APP/PS1 mice following seizures likely contributes to certain physiological responses other than protein synthesis.

### 3.3 Nedd4‐2 Is Associated With Mitochondria and Negatively Regulates Mitochondrial Function

**FIGURE 3:**
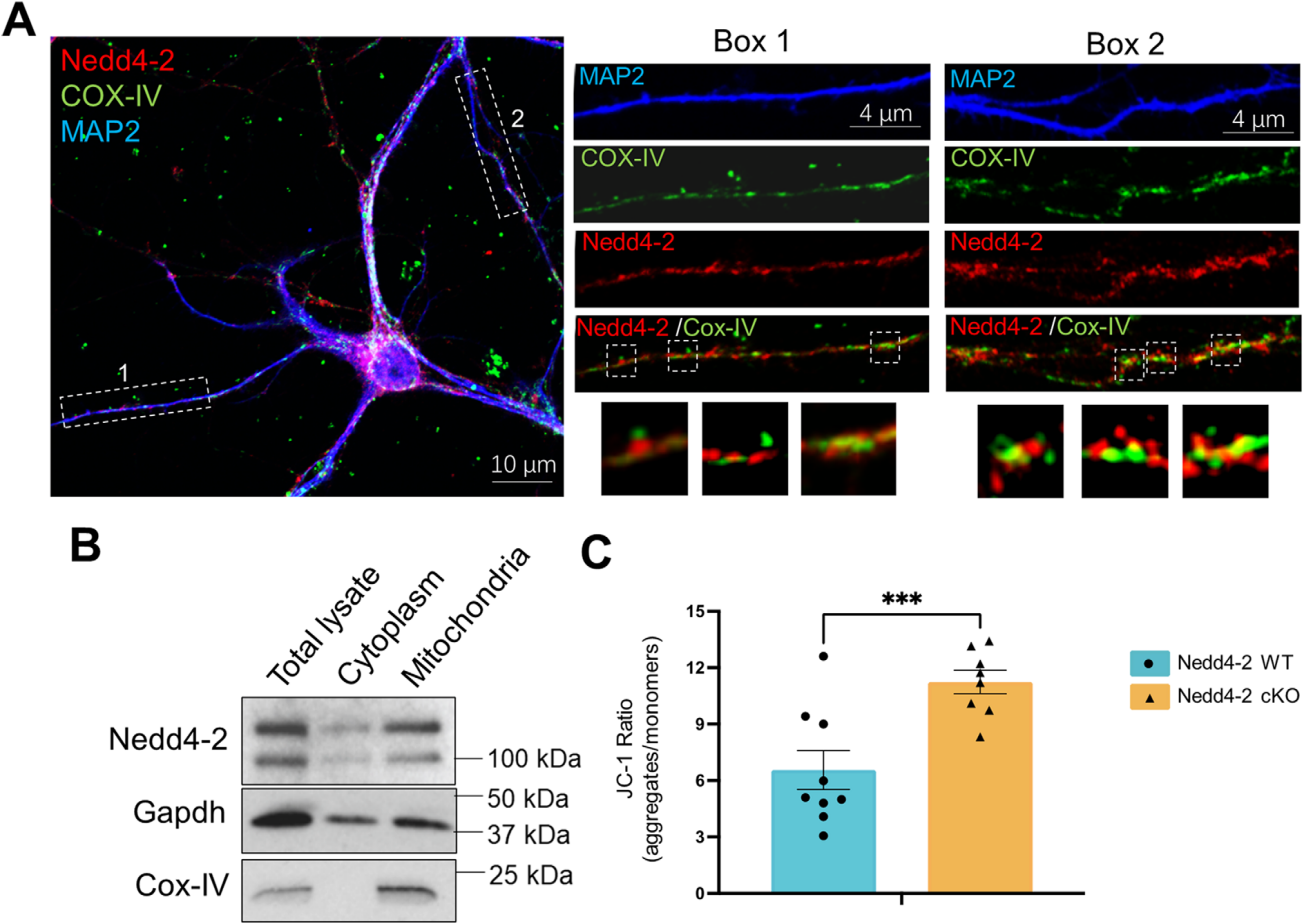
Nedd4‐2 is associated with mitochondria and exhibits a negative function toward mitochondria. (A) Immunocytochemistry images of Nedd4‐2, mitochondrial marker cytochrome C oxidase subunit 4 (COX‐IV) and dendritic marker Microtubule‐associated protein 2 (MAP2) in a cultured WT cortical neuron. Two enlarged dendritic areas are shown on the right. Scale bar: 10 μm. (B) Western blots of Nedd4‐2, COX‐IV, and Glyceraldehyde‐3‐phosphate dehydrogenase (GAPDH) following mitochondrial fractionation using WT mouse brains. (C) Quantification of JC‐1 ratio in Nedd4‐2 WT (*Nedd4‐2* ^*f/f*^ *Emx1* ^−^) and Nedd4‐2 cKO (*Nedd4‐2* ^*f/f*^ *Emx1* ^+^) cortical neuron cultures (*n* = 8–9 independent cultures). Student's *t*‐test was used. Data are represented as mean ± SEM with ***p < 0.001.

Our data showed that seizures differentially alter mitochondrial functions in the brain of WT and APP/PS1 mice. To determine whether Nedd4‐2 is involved, we first asked whether Nedd4‐2 functions in mitochondria. To begin, we performed immunocytochemistry to detect the localization of Nedd4‐2 and mitochondria using cultured WT cortical neurons. As shown in Figure 3A and Figure S2, Nedd4‐2 and mitochondria, which are labeled with the mitochondrial marker cytochrome C oxidase subunit 4 (COX IV), are clustered in similar subcellular regions and their immunofluorescence signals partially overlapped in cultured cortical neurons. When we biochemically isolated mitochondria from the WT mouse brain, we also saw an enrichment of Nedd4‐2 in mitochondrial fractions (Figure 3B). These data suggest an interaction between Nedd4‐2 and mitochondria.

To study whether Nedd4‐2 exerts an influence on mitochondria, we utilized Nedd4‐2 floxed mice (*Nedd4‐2* ^*f/f*^) crossed with *Emx1*‐Cre mice (Jackson Lab) to obtain *Nedd4‐2* ^*f/f‐Emx1‐Cre+*^ (Nedd4‐2 cKO) or *Nedd4‐2* ^*f/f‐Emx1‐Cre‐*^ (Nedd4‐2 WT) mice. *Emx1*‐Cre can confer Nedd4‐2 deletion in forebrain excitatory neurons as early as embryonic day 10.5 (Gorski et al. 2002), and we have previously confirmed an over 80% reduction of Nedd4‐2 in the brains of Nedd4‐2 cKO mice (Zhu et al. 2019). We followed by employing the membrane‐permeant JC‐1 dye to evaluate mitochondria membrane potential of Nedd4‐2 WT and Nedd4‐2 cKO mice. Although JC‐1 is not suitable for in vivo use, it is considered the most sensitive reagent to measure mitochondria membrane potential in vitro. As shown in Figure 3C, we observed a significant elevation of JC‐1 value in cultured primary neurons from Nedd4‐2 cKO cultures, indicating Nedd4‐2's role in reducing mitochondrial membrane potential. Together, our data show that Nedd4‐2 interacts with mitochondria and negatively regulates mitochondrial function.

### 3.4 Nedd4‐2 Regulates the Expression of Various Mitochondrial Proteins Including MFN2

**FIGURE 4:**
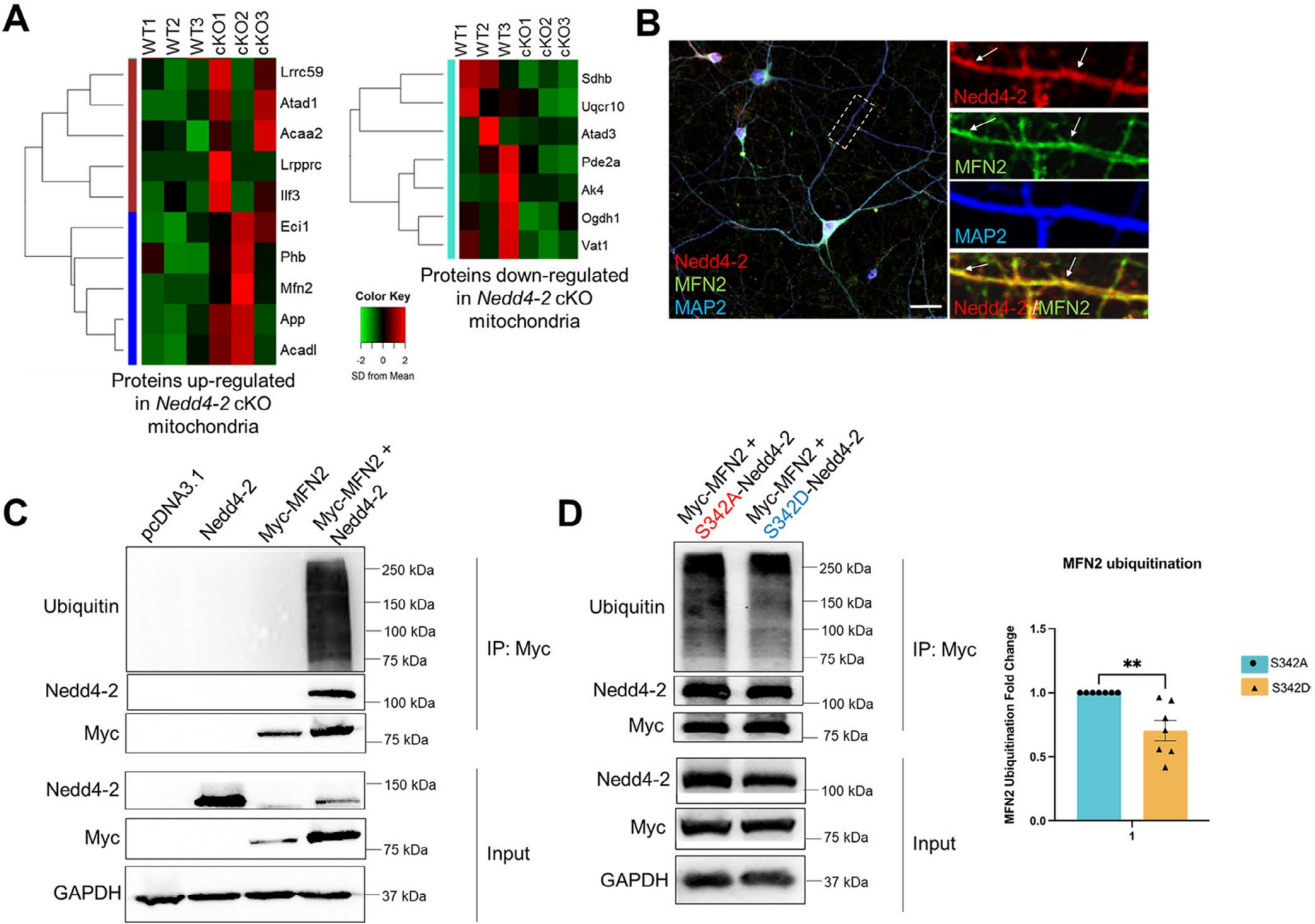
Proteomic screening identifies potential substrates of Nedd4‐2 in the mitochondria. (A) A heat map showing the levels of mitochondrial proteins up‐ or downregulated in Nedd4‐2 cKO brains when compared to Nedd4‐2 WT brains. (B) Immunocytochemistry images of Nedd4‐2, MFN2 and dendritic marker MAP2 in a cultured WT cortical neuron. An enlarged dendritic area is shown on the right. Arrows indicate partial overlapping between Nedd4‐2 and MFN2. Scale bar: 20 μm. (C) Western blots of Nedd4‐2 and ubiquitin (Ub) after immunoprecipitation using anti‐Myc antibody from Human Embryonic Kidney (HEK) 293 cells transfected with a control pcDNA3.1 plasmid, Nedd4‐2 cDNA, Myc‐MFN2 or Myc‐MFN2+ Nedd4‐2 for 24 h. (D) Western blots of Nedd4‐2 and ubiquitin (Ub) after immunoprecipitation using anti‐Myc antibody from HEK 293 cells transfected with Myc‐MFN2 with S342A‐Nedd4‐2 or S342D‐Nedd4‐2 for 24 h. Quantification is shown on the right (*n* = 7 independent cultures). Student's *t*‐test was used. Data are represented as mean ± SEM with **p < 0.01.

To begin exploring the mechanism by which Nedd4‐2 negatively regulates mitochondrial functions, we employed an unbiased proteomic approach using Nedd4‐2 WT and Nedd4‐2 cKO littermate mice to identify novel targets of Nedd4‐2, with the hypothesis that any mitochondrial proteins that were upregulated in the screen may be targets of ubiquitination and subsequent proteasomal degradation mediated by Nedd4‐2 (Table S2). As shown in Figure 4A, we identified 10 mitochondrial proteins that are upregulated and 7 mitochondrial proteins that are downregulated in the brain of Nedd4‐2 cKO mice. Among those proteins, we decided to focus on mitofusin‐2 (or MFN2), a mitochondrial outer membrane protein necessary for maintaining mitochondrial membrane potential (Filadi et al. 2018). As shown in Figure 4B, Nedd4‐2 and MFN2 are clustered in similar subcellular regions of cultured cortical neurons, and their immunofluorescence signals partially overlap. To determine whether MFN2 is a ubiquitination substrate of Nedd4‐2, we co‐expressed Nedd4‐2 and a Myc‐tagged MFN2 into Human Embryonic Kidney (HEK) 293 cells followed by immunoprecipitation with anti‐Myc antibody and western blotting with anti‐Nedd4‐2 antibody to determine MFN2‐Nedd4‐2 interaction or with anti‐ubiquitin antibody to determine MFN2 ubiquitination. We chose HEK 293 cells for this experiment because they do not express endogenous Nedd4‐2 (Eagleman et al. 2021). As shown in Figure 4C, strong interaction between MFN2 and Nedd4‐2 as well as strong MFN2 ubiquitination were observed when MFN2 is co‐expressed with Nedd4‐2. These data suggest that MFN2 is a substrate of Nedd4‐2.

Our data showed that Nedd4‐2 is phosphorylated at S342 in WT mice but stays dephosphorylated in APP/PS1 mice following seizures (Figure 2). Because the substrate specificity of Nedd4‐2 can be mediated by this phosphorylation (Lee et al. 2018), we asked how Nedd4‐2 phosphorylation may affect MFN2 ubiquitination. As shown in Figure 4D, we found that MFN2 exhibits stronger ubiquitination when co‐expressed with a dephospho‐mimetic Nedd4‐2 (S342A‐Nedd4‐2) in contrast to a phospho‐mimetic Nedd4‐2 (S342D‐Nedd4‐2). Altogether, our data suggest that MFN2 can be ubiquitinated and downregulated by Nedd4‐2, especially when it is dephosphorylated.

### 3.5 Nedd4‐2 Downregulates MFN2 Upon Seizures in APP/PS1 Mice

**FIGURE 5:**
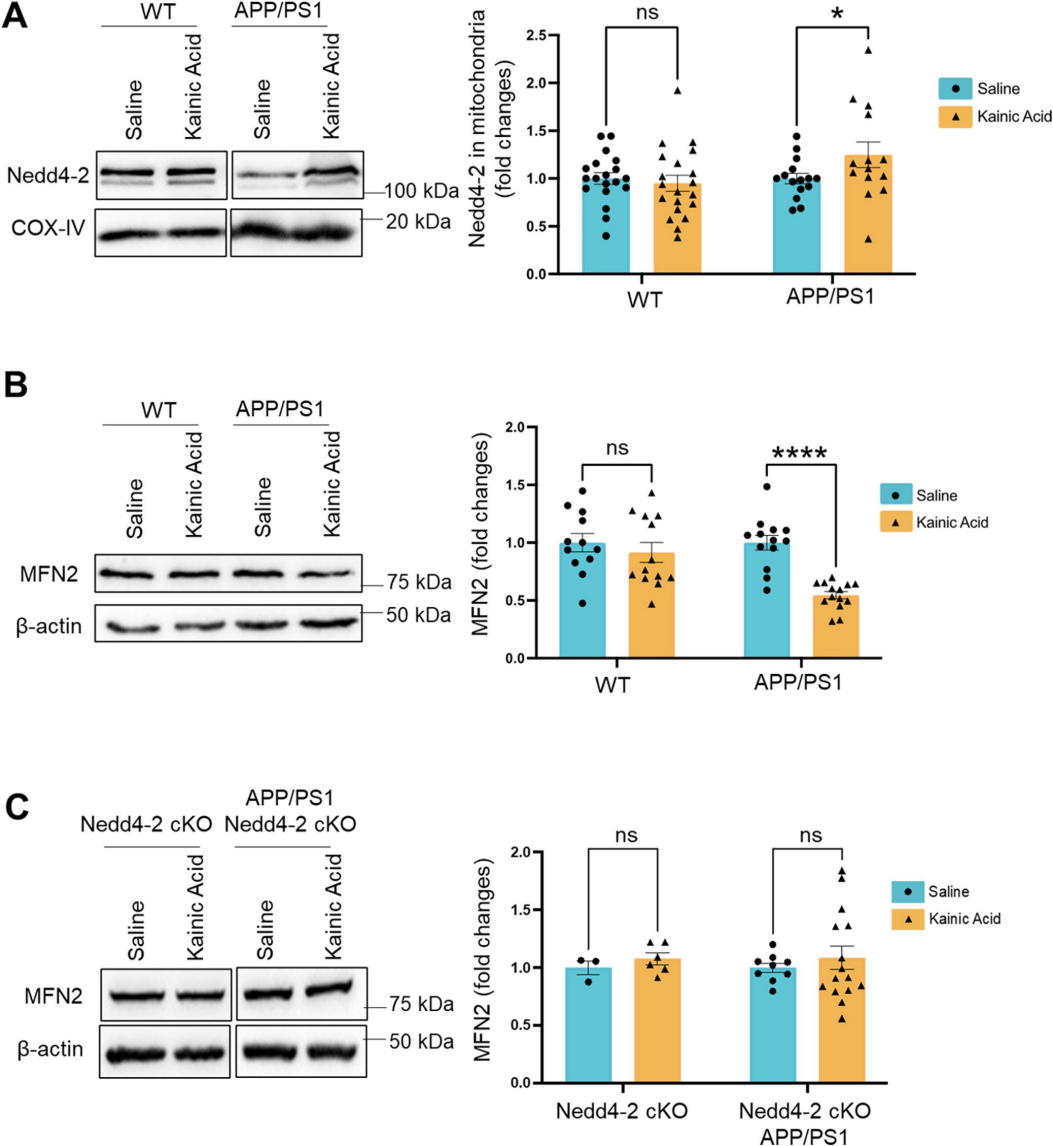
Acute seizures induce Nedd4‐2‐dependent downregulation of MFN2 in APP/PS1 mice. (A) Representative western blots and quantification of Nedd4‐2 and COX‐IV in isolated mitochondria from 10‐week‐old WT and APP/PS1 mice intraperitoneally injected with saline or kainic acid (15 mg/kg) for 2 h (*n* = 14–19 mice per group). (B) Representative western blots and quantification of MFN2 and β‐Actin in brain lysates from 10‐week‐old WT and APP/PS1 mice intraperitoneally injected with saline or kainic acid (15 mg/kg) for 2 h (*n* = 12–14 mice per group). (C) Representative western blots and quantification of MFN2 and β‐Actin in brain lysates from 10‐week‐old Nedd4‐2 cKO and Nedd4‐2 cKO × APP/PS1 mice intraperitoneally injected with saline or kainic acid (15 mg/kg) for 2 h (*n* = 3–13 mice per group). Two‐way ANOVA with Tukey's test was used. Data are represented as mean ± SEM with *p < 0.05, ****p < 0.0001, ns: non‐significant.

**FIGURE 6:**
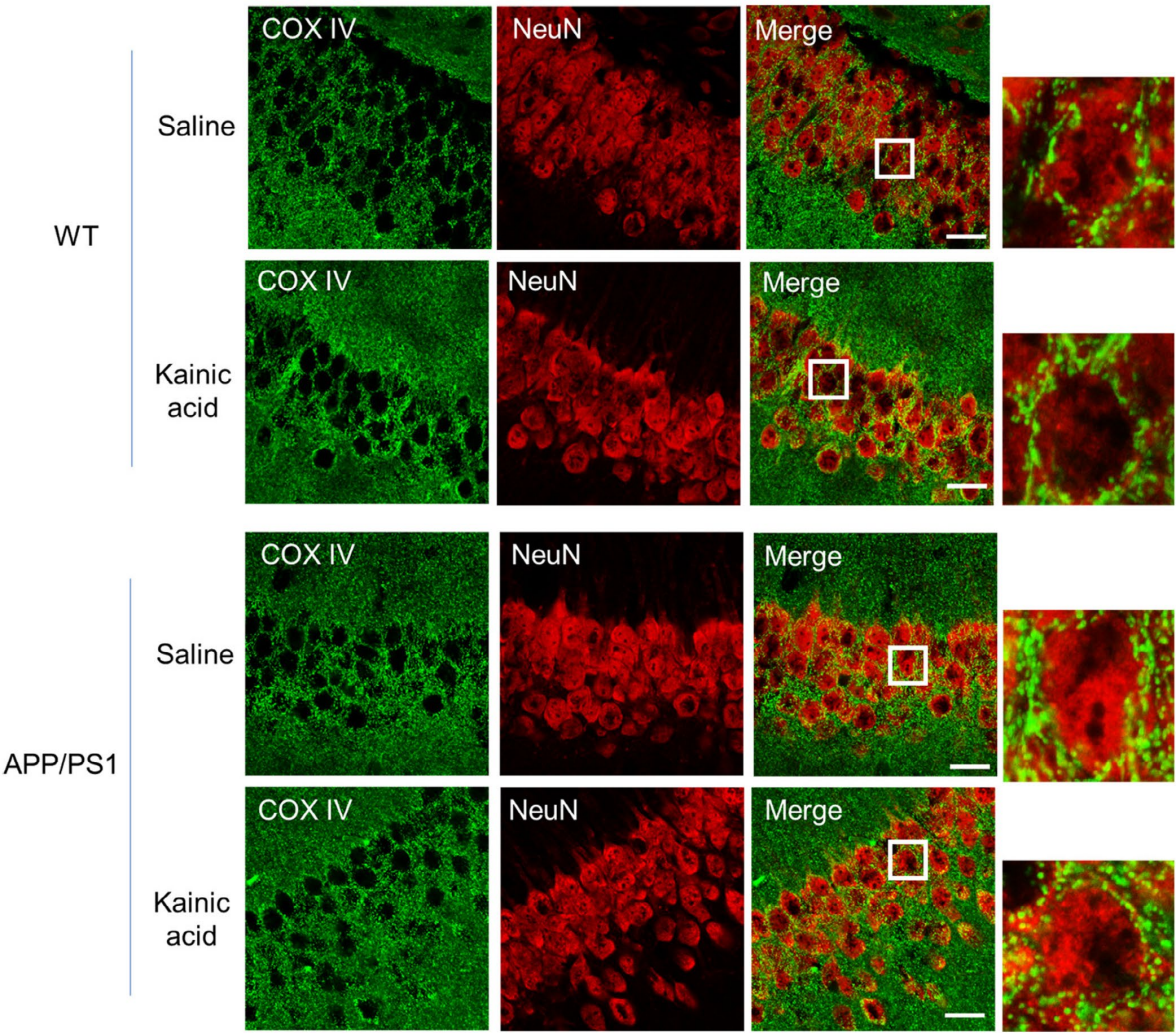
Morphological changes of mitochondria in APP/PS1 mice after seizures. Immunocytochemistry images of COX IV and neuron‐specific nuclear protein (NeuN) from the hippocampal CA1 region of WT or APP/PS1 injected with saline or kainic acid (15 mg/kg) for 2 h. Enlarged images of neuronal cell bodies are shown on the right. Scale bar: 20 μm.

**FIGURE 7:**
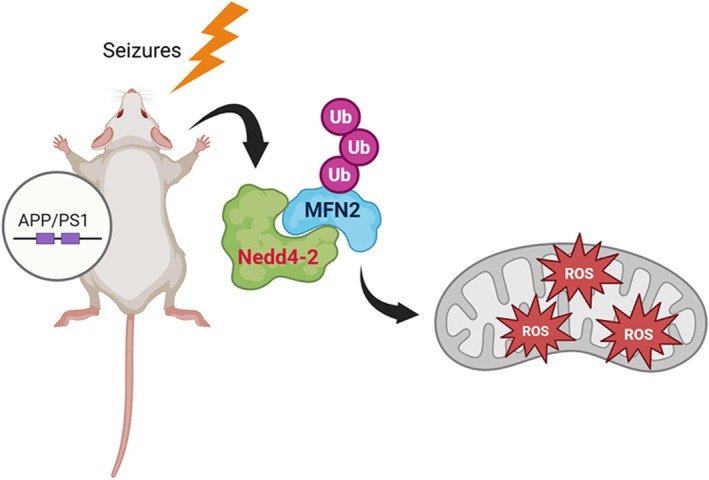
A working hypothesis model. Acute seizures induce an elevation of dephosphorylated Nedd4‐2, leading to ubiquitination and downregulation of MFN2 as well as an elevation of ROS production and potential mitochondrial stress in APP/PS1 mice.

Following the induction of seizures, Nedd4‐2 is elevated but stays dephosphorylated in APP/PS1 mice (Figure 2). Because dephosphorylated Nedd4‐2 can downregulate MFN2, we suspect that Nedd4‐2 downregulates MFN2 in APP/PS1 after seizures. To begin testing this possibility, we intraperitoneally injected WT or APP/PS1 mice with saline or kainic acid (15 mg/kg). Two hours after the injections, we isolated mitochondria and performed western blotting. As shown in Figure 5A, we observed a significant elevation of Nedd4‐2 in mitochondrial fractions in APP/PS1 mice but not WT mice after seizures. Similarly, when measuring the levels of MFN2, we observed a significant reduction of MFN2 only in APP/PS1 mice but not WT mice after seizures (Figure 5B). These results suggest a possibility that Nedd4‐2 mediates MFN2 downregulation in APP/PS1 mice upon seizures. To test this possibility, we crossed APP/PS1 mice with Nedd4‐2 cKO mice to knock down Nedd4‐2 in APP/PS1 mice. As shown in Figure 5C, knocking down Nedd4‐2 impaired seizure‐induced reduction of MFN2 in APP/PS1 mice. Together, our data suggest that, despite basally elevated MFN2 in APP/PS1 mice (Figure S3), seizures induce downregulation of MFN2 in APP/PS1 mice via Nedd4‐2. Because MFN2 is critical for maintaining mitochondrial fusion, we asked whether seizures alter the morphology of mitochondria in APP/PS1 mice. As shown in Figure 6, we observe no obvious changes in mitochondrial morphology in the hippocampus of WT mice, but we observe smaller mitochondria in the hippocampus of APP/PS1 mice after seizures. Altogether, these observations suggest a dysfunction in mitochondria following seizures in APP/PS1 and such an effect is in part contributed by Nedd4‐2 and Nedd4‐2‐mediated ubiquitination (Figure 7).

## 4 Discussion

Alzheimer's disease (AD) is one of the most prevalent neurodegenerative diseases, characterized by neurodegeneration and neuronal death that contribute to cognitive decline and memory impairment. Among the major contributors to AD pathogenesis, the lack of a healthy pool of mitochondria is considered one of the early hallmarks of neurodegeneration (Ahmad and Sachdeva 2022). This is consistent with impaired energy metabolism and elevated oxidative damage frequently observed in AD patients as well as AD animal models (Terada et al. 2020; Troutwine et al. 2022; Yao et al. 2009). A large body of research has also made the connection between altered mitochondrial function and accumulation of toxic Aβ peptides in various AD models (Reddy and Beal 2008). For example, studies have demonstrated that intracellular Aβ can either localize in mitochondrial membrane and disrupt the continuity of the electron transport chain (Manczak et al. 2006) or indirectly induce intracellular calcium overload, leading to enhanced mitochondria membrane depolarization and elevated production of reactive oxygen species (ROS) (Calvo‐Rodriguez et al. 2020). However, it remains largely unknown about the connection between mitochondrial defects and common comorbidities in AD. In our current study, we found that acute seizures reduce ROS in WT but not APP/PS1 mice (Figure 6). Because chronic epilepsy is known to promote ROS production (Eastman et al. 2020), a reduction of ROS following acute seizures in WT mice suggests a likelihood of counterresponse to hyperexcitability‐induced cellular stress. However, since our kainic acid–induced seizure model does not induce chronic epilepsy, it would be important to characterize how WT and APP/PS1 mice differently modulate reactive oxygen species (ROS) production during chronic epilepsy. On the other hand, since we observed impaired ROS production in young APP/PS1 mice, it suggests that the cellular stress response is likely altered in early‐stage AD, making the brain more prone to seizures‐induced damage. Because elevated cellular stress could also occur following other comorbidities in AD, such as hemorrhagic stroke (Waziry et al. 2020), insufficient or altered stress response may exacerbate neurodegeneration and brain damage in AD patients with such comorbidities. Future research into altered cellular stress response in early‐stage AD may reveal the mechanisms underlying accelerated neurodegeneration in AD with certain comorbidities and foster future therapeutic development.

Nedd4‐2 belongs to the Nedd4 family of E3 ubiquitin ligases (Yang and Kumar 2010) but is the only member encoded by an epilepsy‐associated gene (Allen et al. 2013). Nedd4‐2 has a high affinity toward interacting and ubiquitinating membrane‐associated proteins and ion channels, leading to its function in regulating basal seizure threshold (Zhu et al. 2017, 2019). Our previous study has shown that Nedd4‐2 interacts with membrane organelles such as endoplasmic reticulum (Lodes et al. 2022), and our current study observed Nedd4‐2 is associated with mitochondria. Interestingly, we found that Nedd4‐2 is highly phosphorylated following seizures in WT mice but remains dephosphorylated in APP/PS1 mice. Because the substrate specificity of Nedd4‐2 is largely regulated by its phosphorylation status, our data suggest that many substrates of Nedd4‐2 are likely dysregulated in APP/PS1 mice. Since Nedd4‐2 is dephosphorylated following seizures in APP/PS1 mice, promoting Nedd4‐2 phosphorylation may have beneficial effects in AD. Because our published work has shown that Akt mediates phosphorylation of Nedd4‐2 in cortical excitatory neurons (Lee et al. 2018), whereas others have found that Akt is downregulated in AD mouse models (Chen et al. 2013; Mana et al. 2019), it suggests that modulating Akt activity may improve mitochondrial functions through Nedd4‐2 phosphorylation. This idea necessitates future experiments to explore and validate.

Mitofusin 2 (MFN2) is an outer mitochondrial membrane GTPase that has various functions, including regulating mitochondrial fusion, structural integrity, membrane potential, and mitochondrial intracellular trafficking (Filadi et al. 2018). In AD, studies have observed a reduction of MFN2 in the frontal cortex of patients as well as in the hippocampus of postmortem patient samples (Manczak et al. 2011; Wang et al. 2009). In animal models, studies have also shown reduced expression of MFN2 following the expression of Aβ (Xu et al. 2018). Using a neuron‐specific knockout model of Nedd4‐2 in mice (Nedd4‐2 cKO), our unbiased proteomic screening has identified a group of mitochondrial proteins as potential substrates of Nedd4‐2. Using an in vitro system, we confirmed that MFN2 is a ubiquitination substrate of Nedd4‐2 particularly when it is dephosphorylated. Because Nedd4‐2‐induced MFN2 downregulation appears to only occur in APP/PS1 mice upon acute seizure induction based on our data, it supports our overall conclusion that mitochondria are prone to seizure‐induced damage in young APP/PS1 mice. What remains to be determined is whether this Nedd4‐2‐MFN2 axis can occur without seizure‐like provocation in late‐phase AD. Our published work has shown that Akt mediates phosphorylation of Nedd4‐2 in hippocampal neurons (Lee et al. 2018), whereas others have found that Akt is downregulated in aged AD mouse models (Chen et al. 2013; Mana et al. 2019). This information suggests that Nedd4‐2 may be basally dephosphorylated in late‐stage AD, leading to enhanced ubiquitination and downregulation of MFN2. As stated previously, AD‐associated mitochondrial defects are complex and remain largely elusive. Further research is needed for a clear picture of altered mitochondrial functions across different animal models and patients of AD, different stages of disease progression and different clinical or comorbid phenotypes.

## Author Contributions

**Yingxin Wang:** conceptualization, investigation, formal analysis, writing. **Jiuhe Zhu:** investigation, formal analysis. **Simon Lizarazo:** investigation. **Kwan Young Lee:** investigation, formal analysis. **Olivia Wong:** investigation, formal analysis. **Sophia Azim:** investigation, formal analysis. **Yeeun Yook:** investigation, formal analysis. **Vipendra Kumar:** investigation. **Nien‐Pei Tsai:** conceptualization, funding acquisition, writing.

## Funding

This work was supported by the Alzheimer's Association (AARG‐23‐1149282); National Institute on Aging (R21AG071278 and R21AG089491).

## Conflicts of Interest

The authors declare no conflicts of interest.

## Supporting information


**Figure S1:** Acute seizures do not alter the expression of other mitochondrial markers in WT or APP/PS1 mice. Representative western blots and quantification of Translocase of Outer Mitochondrial Membrane 20 (TOM20), Voltage‐Dependent Anion Channel (VDAC), mitochondria marker COX IV and β‐actin in purified mitochondria from 10‐week‐old wild‐type (WT) and APP/PS1 mice intraperitoneally injected with saline or kainic acid (15 mg/kg) for 2 h (*n* = 18–21 mice per group). Two‐way ANOVA with Tukey's test was used. Data are represented as mean ± SEM with ns: nonsignificant.
**Figure S2:** An additional image showing partial colocalization between Nedd4 2 and COX IV. Immunocytochemistry images of Nedd4‐2 and mitochondrial marker cytochrome C oxidase subunit 4 (COX‐IV) in a cultured WT cortical neuron. An enlarged dendritic area was shown on the right. Scale bar: 10 μm.
**Figure S3:** MFN2 is basally elevated in the total brain lysates of APP/PS1 mice at 10 weeks of age. Representative western blots of Mitofusin 2 (MFN2) and β‐actin in total brain lysates from 10‐week‐old WT and APP/PS1 mice (left) and quantification (right) (*n* = 12 mice per group). Student's t‐test was used. Data are represented as mean ± SEM with *p < 0.05.
**Table S1:** Full Statistical Reports for all data in this study. Uncropped Full Images of Western Blotting Results.
**Table S2:** Label‐free proteomics screening identifies proteins that are up‐ or down‐regulated in Nedd4‐2 cKO brains.

## Data Availability

The data that support the findings of this study are available from the corresponding author upon reasonable request.
