# Supplementary material for: E3 Ubiquitin Ligase Nedd4‐2 Exacerbates Seizure‐Induced Mitochondrial Defects in an Alzheimer's Disease Mouse Model
Source: J Neurochem. 2026 Apr 14;170(4):e70440. doi: 10.1111/jnc.70440 (PMC13080284; doi:10.1111/jnc.70440)

Supplemental Information

E3 Ubiquitin Ligase Nedd4-2 Exacerbates Seizure-induced Mitochondrial Defects in an Alzheimer's Disease Mouse Model

Yingxin Wang[1], Jiuhe Zhu[1], Simon Lizarazo[1], Kwan Young Lee[1], Olivia Wong[1], Sophia Azim[1], Yeeun Yook[1], Vipendra Kumar[1], and Nien-Pei Tsai[1,2,3,4,*]

[1]Department of Molecular and Integrative Physiology, School of Molecular and Cellular Biology, University of Illinois at Urbana-Champaign, Urbana, IL 61801, USA

[2]Neuroscience Program, University of Illinois at Urbana-Champaign, Urbana, IL 61801, USA

[3]Beckman Institute, University of Illinois at Urbana-Champaign, Urbana, IL 61801, USA

[4]Cancer Center at Illinois, University of Illinois at Urbana-Champaign, Urbana, IL 61801, USA

*CORRESPONDENCE: Nien-Pei Tsai, Ph.D.

407 South Goodwin Ave., Urbana, IL 61801, USA

Tel: 217-244-5620 Fax: 217-333-1133

Email: nptsai@illinois.edu

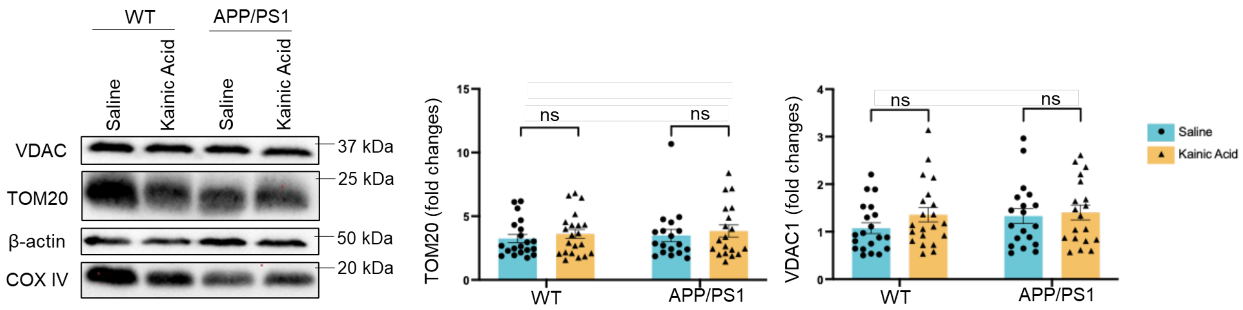

Supplemental Figure S1: Acute seizures do not alter the expression of other mitochondrial markers in WT or APP/PS1 mice. Representative western blots and quantification of Translocase of Outer Mitochondrial Membrane 20 (TOM20), Voltage-Dependent Anion Channel (VDAC), mitochondria marker COX IV and β-actin in purified mitochondria from 10-week-old wild-type (WT) and APP/PS1 mice intraperitoneally injected with saline or kainic acid (15 mg/kg) for 2 hours (n = 18-21 mice per group). Two-way ANOVA with Tukey's test was used. Data are represented as mean ± SEM with ns: non-significant.

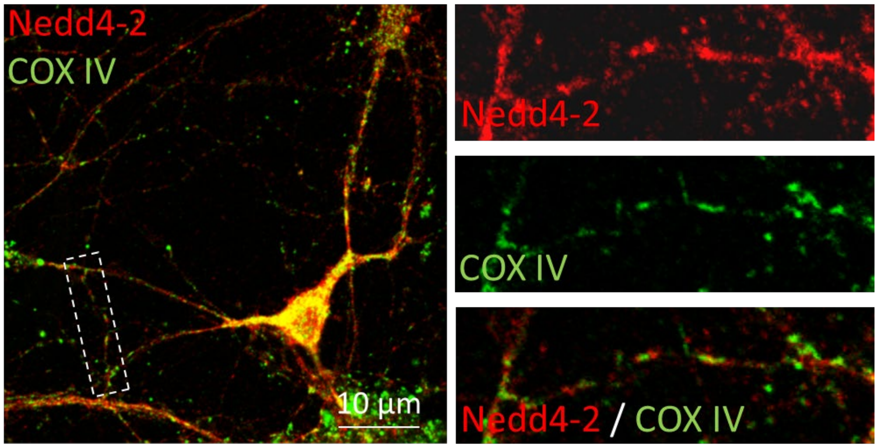

Supplemental Figure S2: An additional image showing partial colocalization between Nedd4-2 and COX IV. Immunocytochemistry images of Nedd4-2 and mitochondrial marker cytochrome C oxidase subunit 4 (COX-IV) in a cultured WT cortical neuron. An enlarged dendritic area was shown on the right. Scale bar: 10 µm.

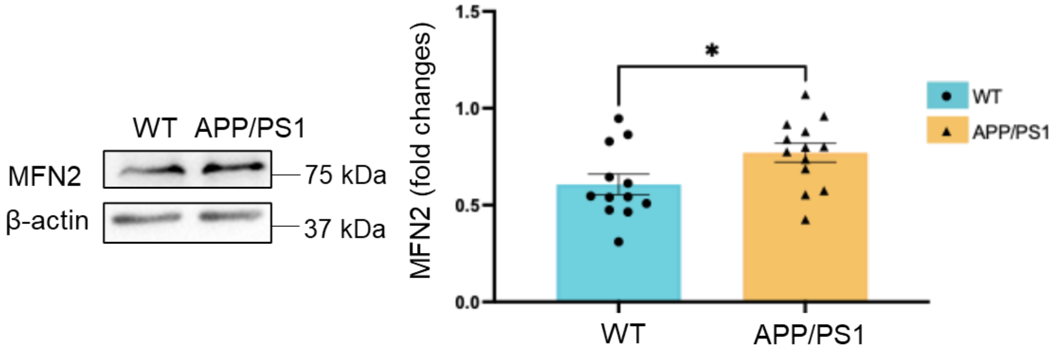

Supplemental Figure S3: MFN2 is basally elevated in the total brain lysates of APP/PS1 mice at 10 weeks of age. Representative western blots of Mitofusin 2 (MFN2) and β-actin in total brain lysates from 10-week-old WT and APP/PS1 mice (left) and quantification (right) (n = 12 mice per group). Student's *t*-test was used. Data are represented as mean ± SEM with *P<0.05.

Supplemental Table S1: Full Statistical Reports for all data in this study.

Two-way ANOVA analyses for Figure 1A.

Result	ANOVA table	F(DFn, DFd)	p value
Western blot NRF1 fold change	Kainic acid treatment	F(1, 63) = 4.040	p = 0.487
	Genotype	F(1, 63) = 1.075	p = 0.3038
	Interaction	F(1, 63) = 1.075	p = 0.3038

Multiple comparisons		p value
WT	Saline vs. Kainic acid	p = 0.0335
APP/PS1	Saline vs. Kainic acid	p = 0.4976

Two-way ANOVA analyses for Figure 1B.

Result	ANOVA table	F(DFn, DFd)	p value
ROS fold change	Kainic acid treatment	F(1, 38) = 8.151	p = 0.0069
	Genotype	F(1, 40) = 1.273	p = 0.2660
	Interaction	F(1, 38) = 1.313	p = 0.2590

Multiple comparisons		p value
WT	Saline vs. Kainic acid	p = 0.0067
APP/PS1	Saline vs. Kainic acid	p = 0.2402

Two-way ANOVA analyses for Figure 2A.

Result	ANOVA table	F(DFn, DFd)	p value
Western blot Nedd4-2 fold change	Treatment	F(1, 27) = 10.99	p = 0.0026
	Genotype	F(1, 27) = 3.712	p = 0.0646
	Interaction	F(1, 27) = 3.712	p = 0.0646
Western blot p-Nedd4-2 fold change	Treatment	F(1, 10) = 8.220	p = 0.0168
	Genotype	F(1, 16) = 3.634	p = 0.0747
	Interaction	F(1, 10) = 8.213	p = 0.0168

Multiple comparisons		p value
Nedd4-2 WT	Saline vs. Kainic acid	p = 0.6173
Nedd4-2 APP/PS1	Saline vs. Kainic acid	p = 0.0121
p-Nedd4-2 WT	Saline vs. Kainic acid	p = 0.0012
p-Nedd4-2 APP/PS1	Saline vs. Kainic acid	p = 0.7971
p-Nedd4-2 Kainic acid	WT vs. APP/PS1	p = 0.0013

Two-way ANOVA analyses for Figure 2B.

Result	ANOVA table	F(DFn, DFd)	p value
Puromycin labeling fold change	Treatment	F(1, 27) = 50.70	p < 0.0001
	Genotype	F(1, 27) = 0.7359	p = 0.3985
	Interaction	F(1, 27) = 0.7359	p = 0.3985

Multiple comparisons		p value
WT	Saline vs. Kainic acid	p = 0.0003
APP/PS1	Saline vs. Kainic acid	p < 0.0001

Paired t-Test analyses for Figure 3C.

Result	t-Test table	T(t, df)	p value
JC-1 Ratio	Genotype	t = 6.330<br>df = 7	p = 0.0004

Paired t-Test analyses for Figure 4D.

Result	t-Test table	T(t, df)	p value
Western blot MFN2 ubiquitination fold change	Mutant Nedd4-2	t = 3.726<br>df = 6	p = 0.0098

Two-way ANOVA analyses for Figure 5A.

Result	ANOVA table	F(DFn, DFd)	p value
Western blot Nedd4-2 in mitochondria	Treatment	F(1, 19) = 1.792	p = 0.1965
	Genotype	F(1, 19) = 3.150	p = 0.0919
	Interaction	F(1, 7) = 3.637	p = 0.0982

Multiple comparisons		p value
WT	Saline vs. Kainic acid	p = 0.6669
APP/PS1	Saline vs. Kainic acid	p = 0.0417

Two-way ANOVA analyses for Figure 5B.

Result	ANOVA table	F(DFn, DFd)	p value
Western blot MFN2 fold change	Treatment	F(1, 48) = 16.48	p = 0.0002
	Genotype	F(1, 48) = 7.738	p = 0.0077
	Interaction	F(1, 48) = 7.738	p = 0.0077

Multiple comparisons		p value
WT	Saline vs. Kainic acid	p = 0.9431
APP/PS1	Saline vs. Kainic acid	p < 0.0001

Two-way ANOVA analyses for Figure 5C.

Result	ANOVA table	F(DFn, DFd)	p value
Western blot MFN2 fold change with Nedd4-2 depletion	Treatment	F(1, 22) = 0.1626	p = 0.6906
	Genotype	F(1, 7) = 0.1409	p = 0.7185
	Interaction	F(1, 7) = 0.1409	p = 0.7185

Multiple comparisons		p value
Nedd4-2 cKO	Saline vs. Kainic acid	p = 0.9474
Nedd4-2 cKO APP/PS1	Saline vs. Kainic acid	p = 0.4704

Two-way ANOVA analyses for TOM20 in Supplemental Figure S1.

Result	ANOVA table	F(DFn, DFd)	p value
Western blot TOM20 in mitochondria	Treatment	F(1, 74) = 0.7916	p = 0.3855
	Genotype	F(1, 74) = 0.3326	p = 0.5659
	Interaction	F(1, 74) = 0.0001303	p = 0.9909

Multiple comparisons		p value
WT	Saline vs. Kainic acid	p = 0.8830
APP/PS1	Saline vs. Kainic acid	p = 0.8941

Two-way ANOVA analyses for VDAC1 in Supplemental Figure S1.

Result	ANOVA table	F(DFn, DFd)	p value
Western blot VDAC1 in mitochondria	Treatment	F(1, 75) = 1.099	p = 0.2979
	Genotype	F(1, 75) = 1.492	p = 0.2257
	Interaction	F(1, 75) = 0.5065	p = 0.4789

Multiple comparisons		p value
WT	Saline vs. Kainic acid	p = 0.5073
APP/PS1	Saline vs. Kainic acid	p = 0.9847

Paired t-Test analyses for baseline MFN2 in Supplemental Figure S3.

Result	t-Test table	T(t, df)	p value
Baseline MFN2	Genotype	t = 2.251<br>df = 23	p = 0.0342

Uncropped Full Images of Western Blotting Results

Figure 1

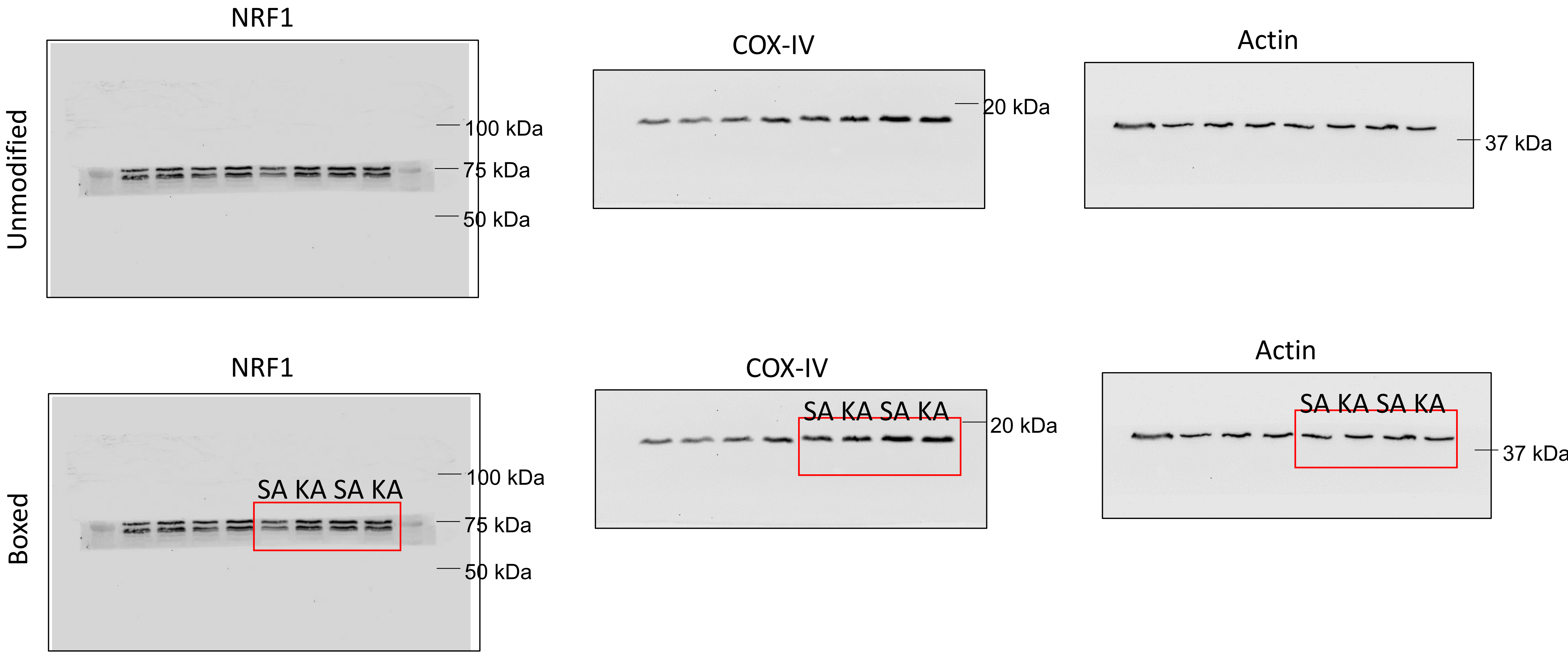

Figure 2A_Nedd4-2

Unmodified

Nedd4-2 from WT mice

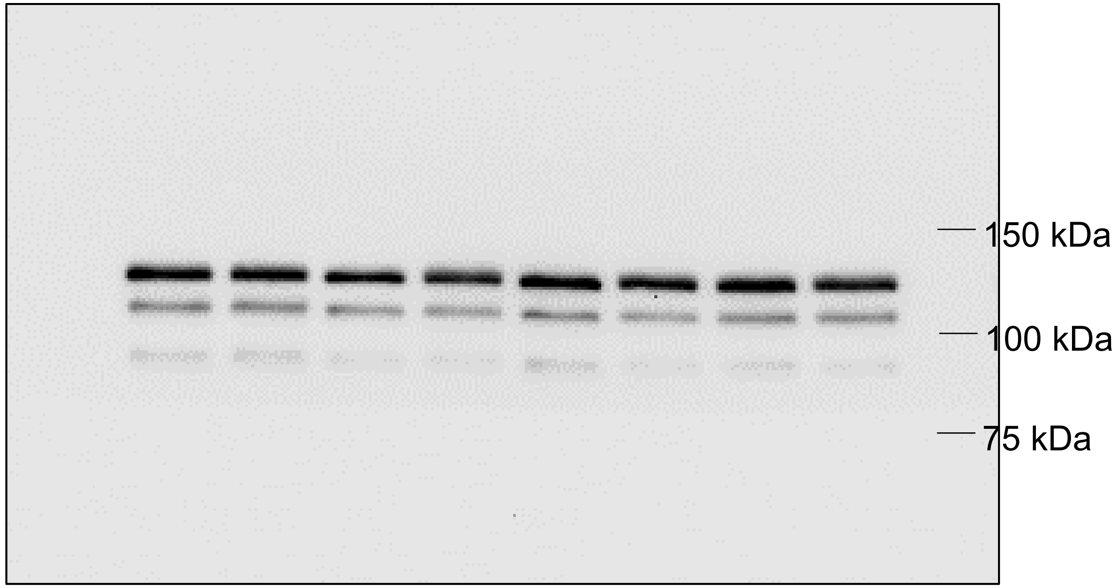

Nedd4-2 from APP/PS1 mice

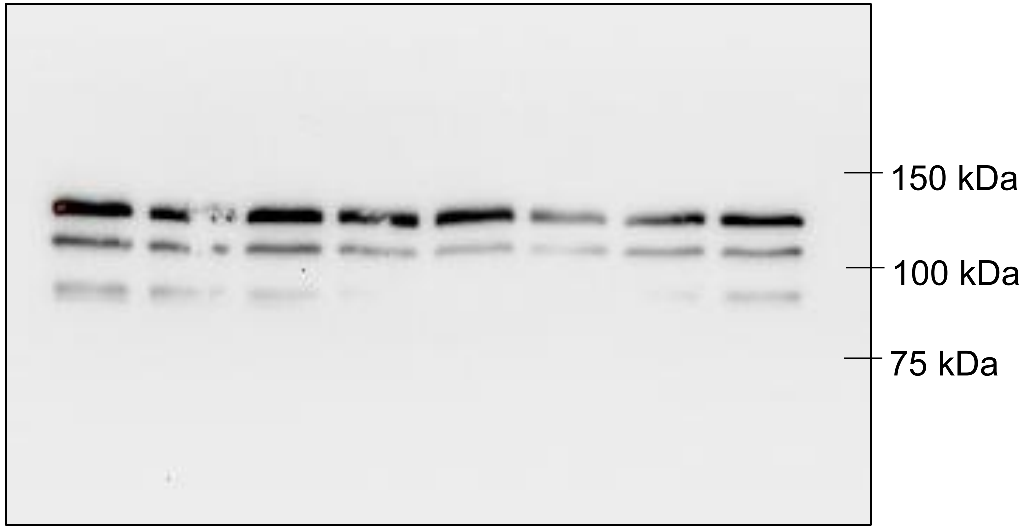

Boxed

Nedd4-2 from WT mice

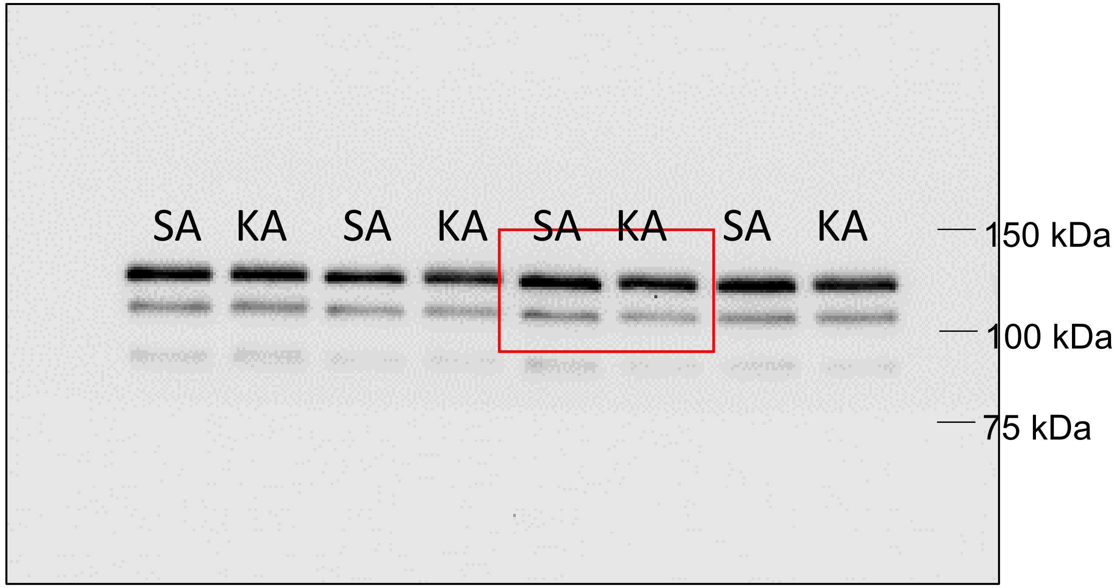

Nedd4-2 from APP/PS1 mice

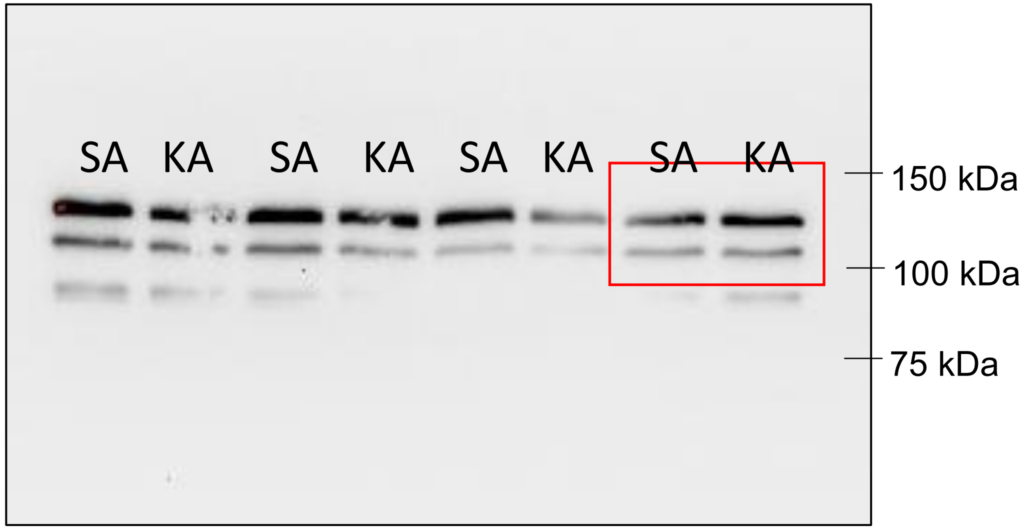

Figure 2A_p-Nedd4-2

Unmodified

p-Nedd4-2 from WT mice

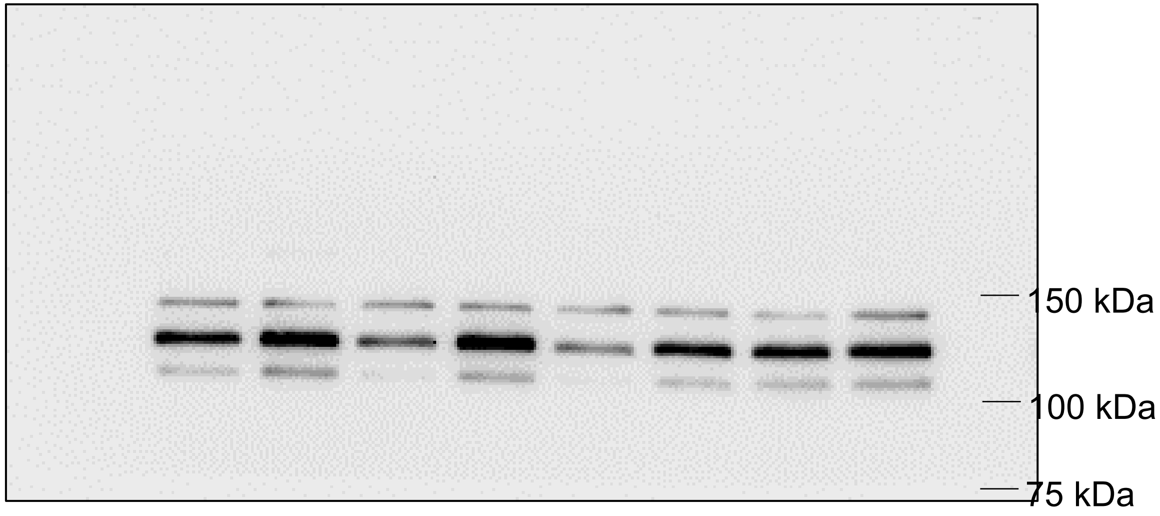

p-Nedd4-2 from APP/PS1 mice

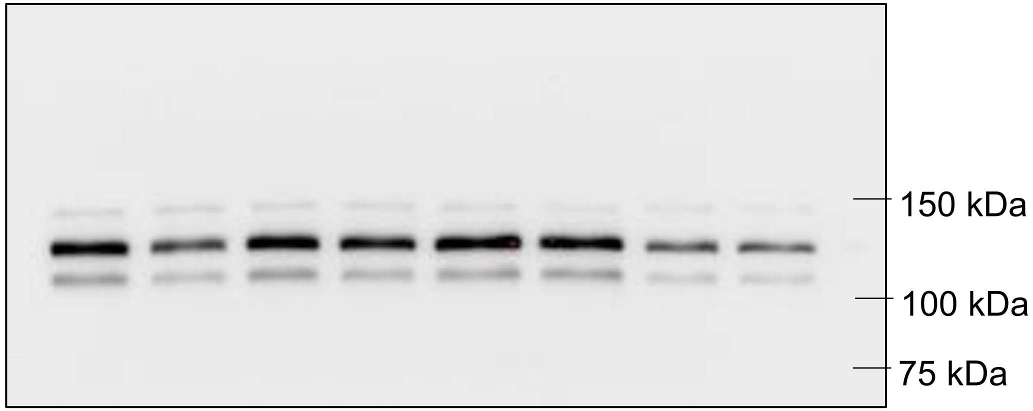

Boxed

p-Nedd4-2 from WT mice

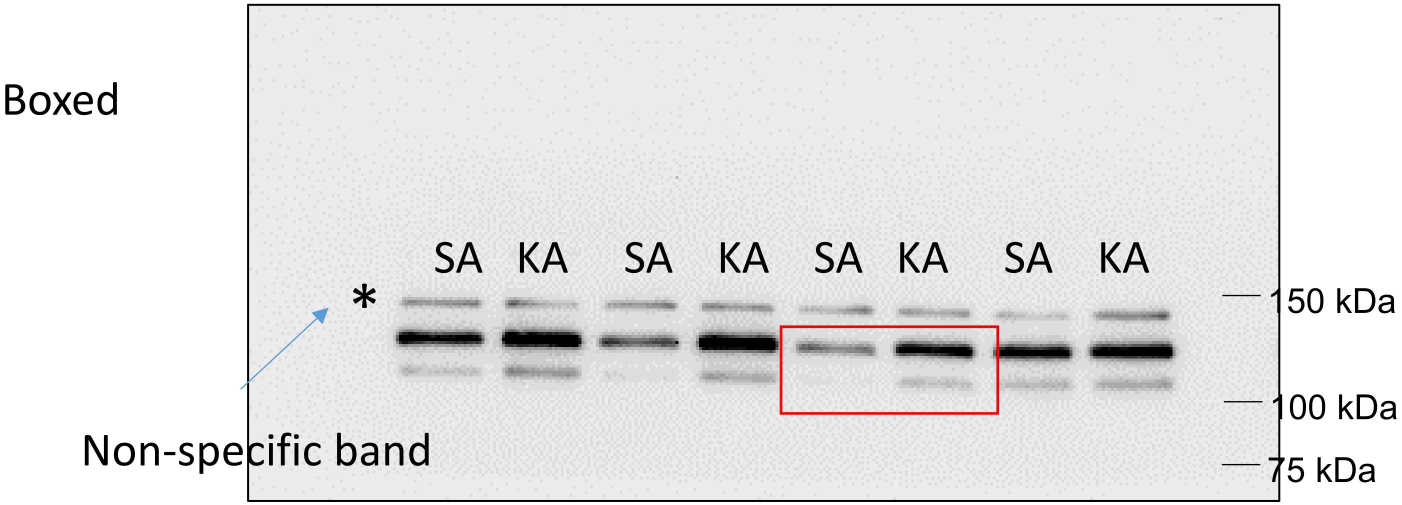

p-Nedd4-2 from APP/PS1 mice

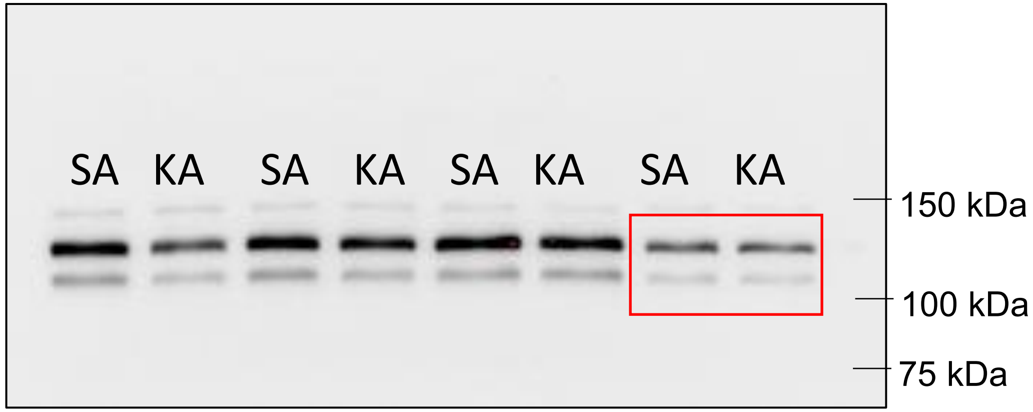

Figure 2A_Actin

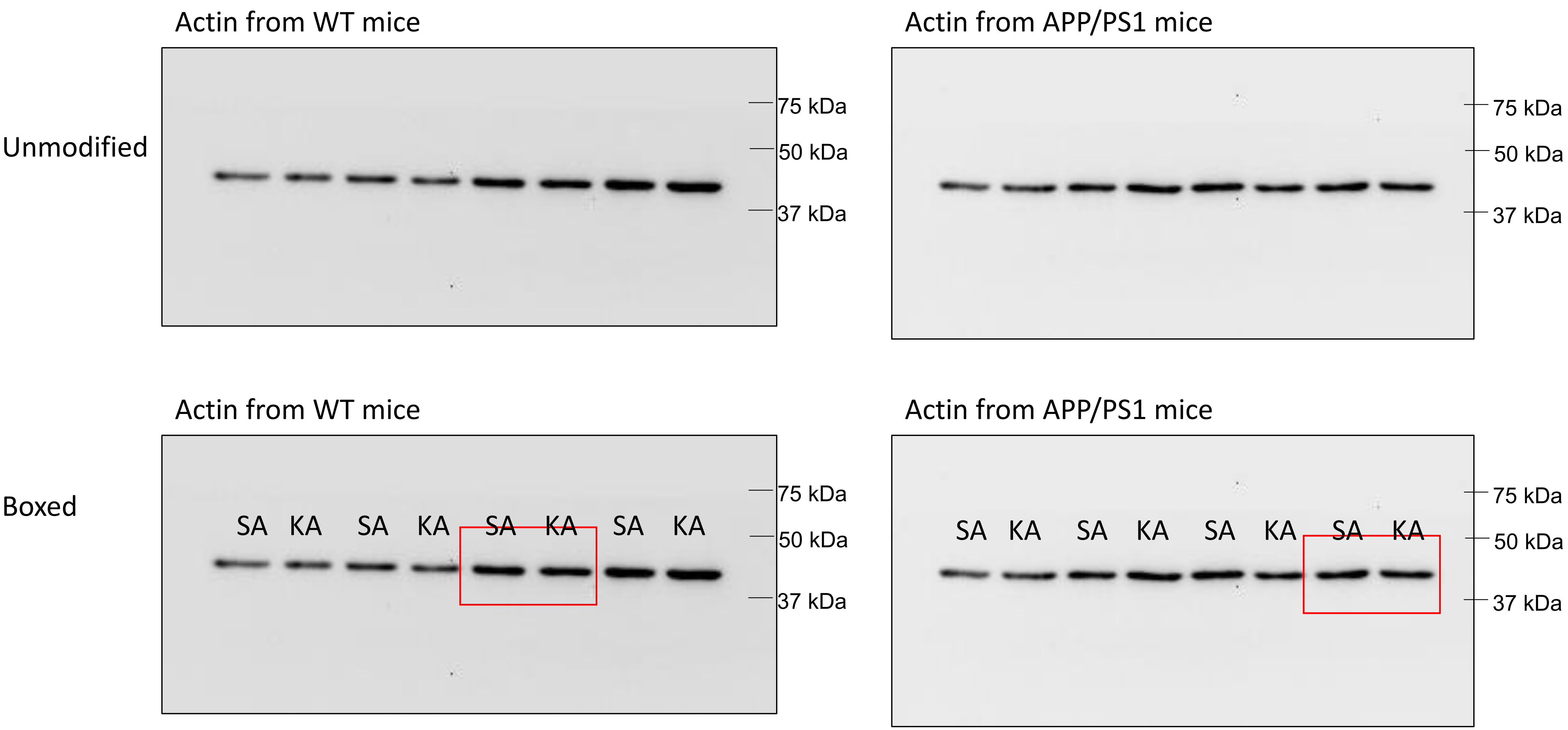

Figure 2B_Puromycin

Unmodified

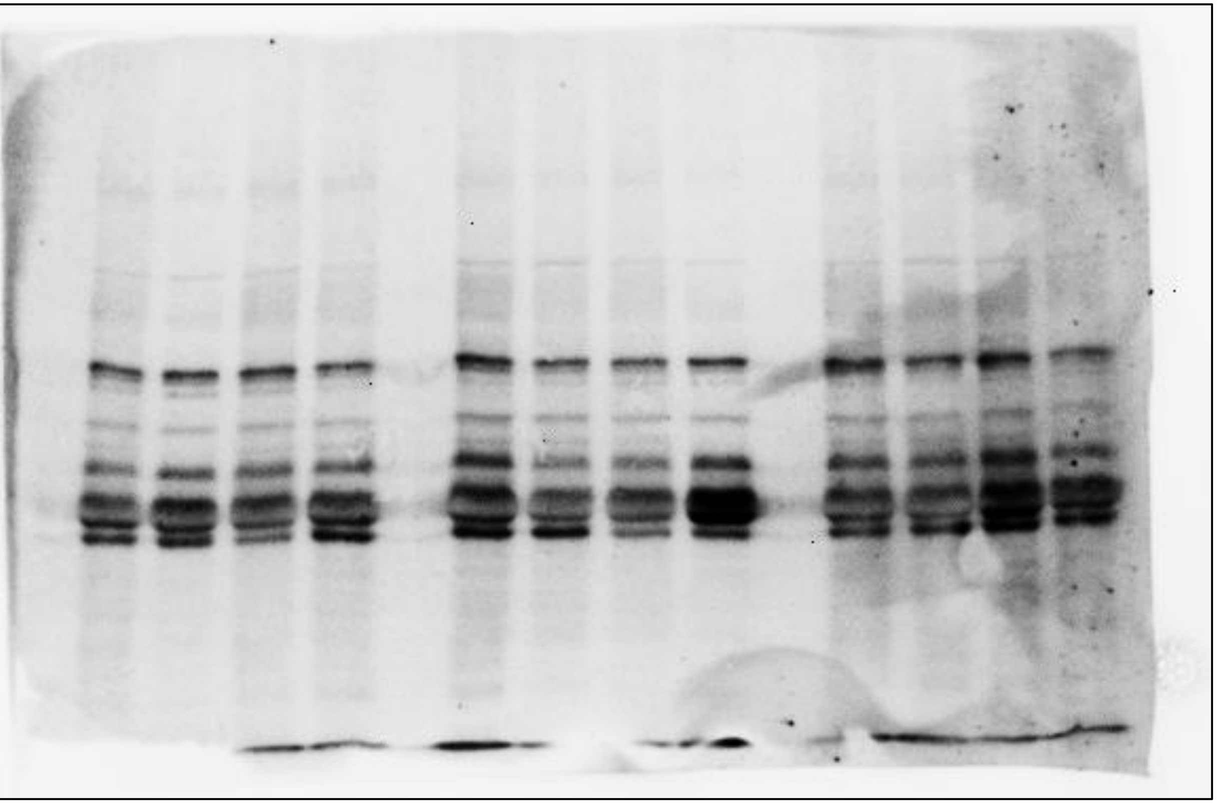

Boxed

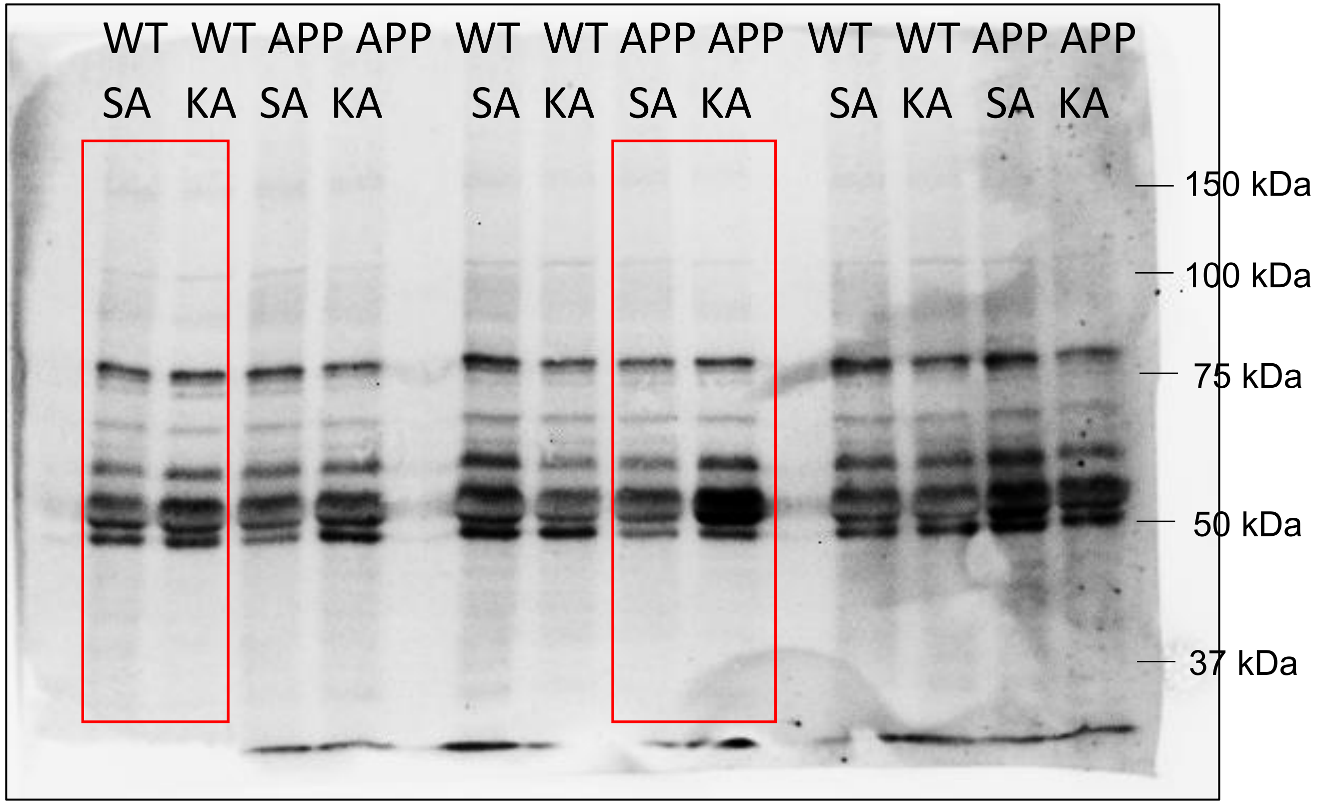

Figure 2B_Actin

Unmodified

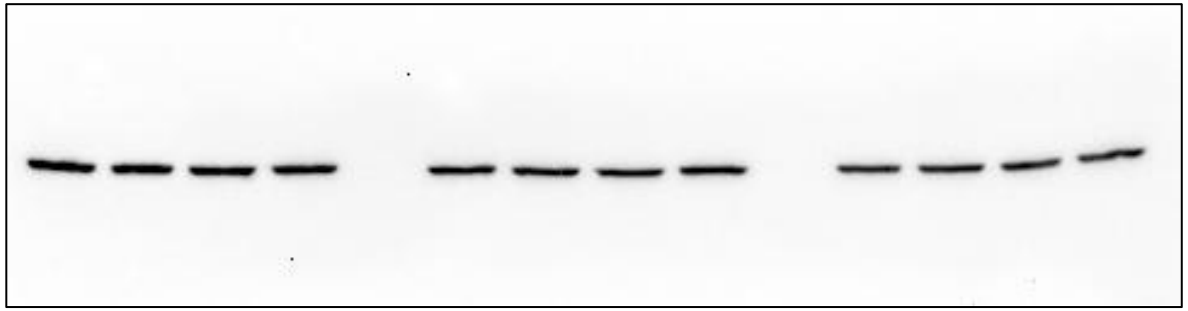

Boxed

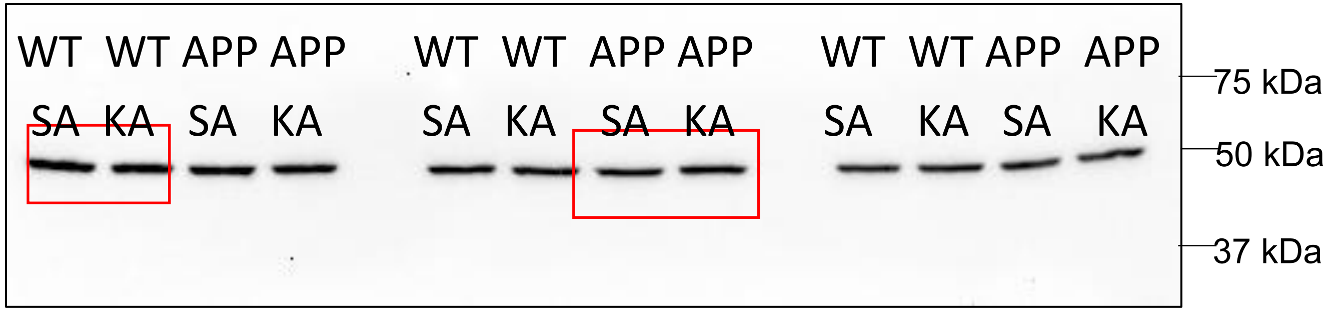

Figure 3B

Nedd4-2

Unmodified

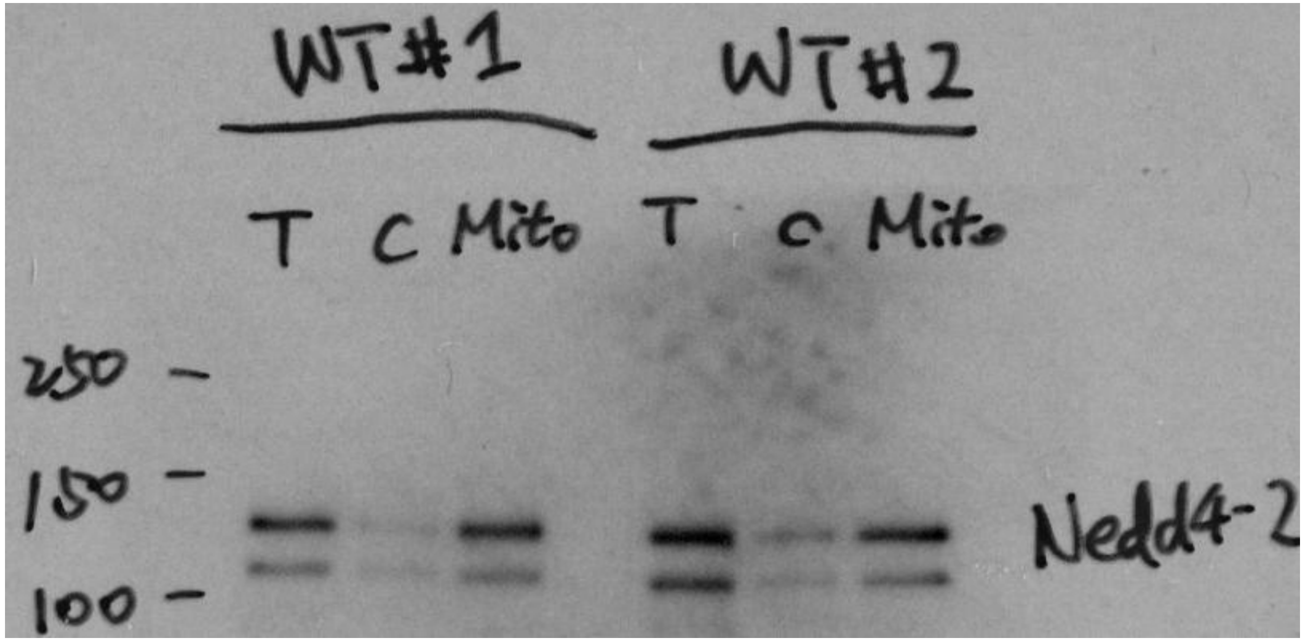

Boxed

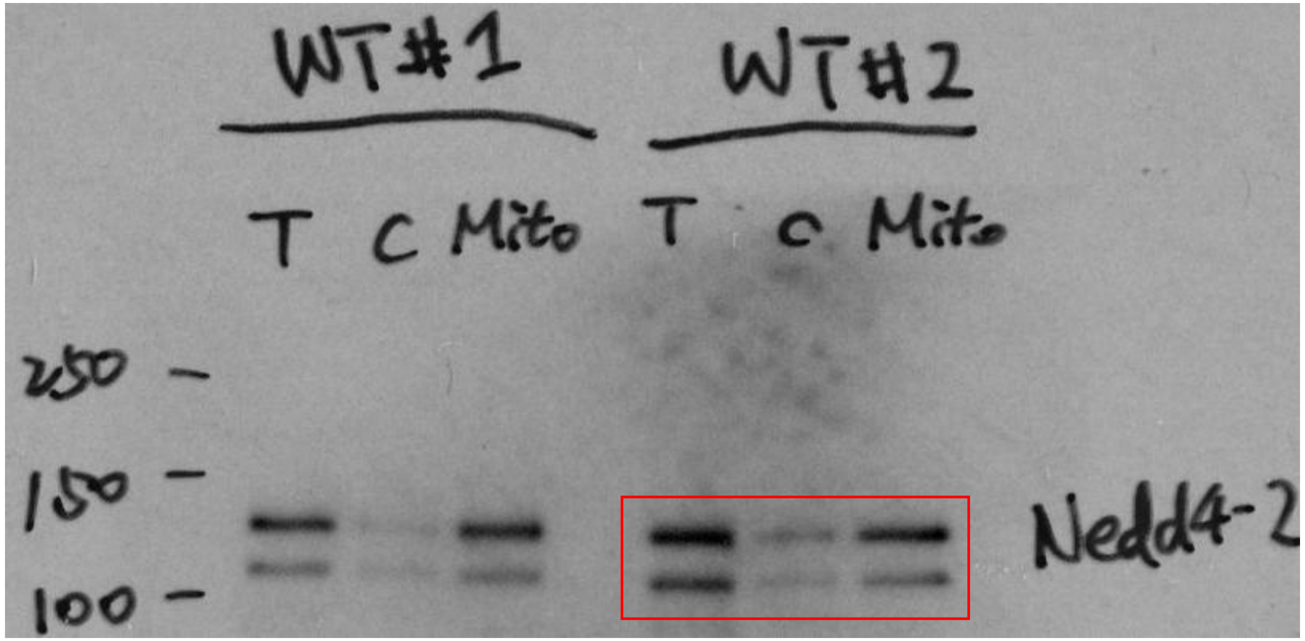

Figure 3B

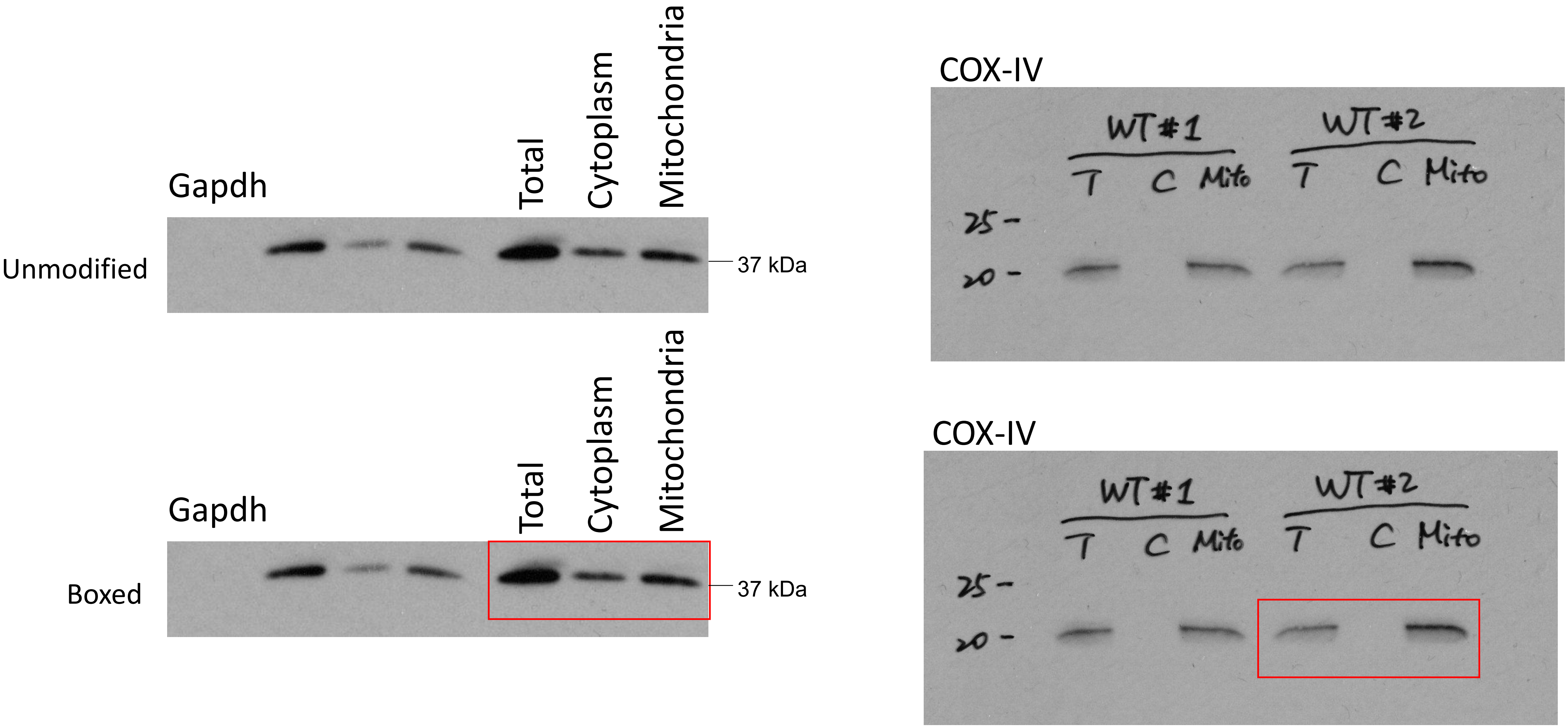
Gapdh
Total
Cytoplasm
Mitochondria
Unmodified
37 kDa
Gapdh
Total
Cytoplasm
Mitochondria
Boxed
37 kDa
COX-IV
WT#1
WT#2
T
C
Mito
T
C
Mito
25
20
COX-IV
WT#1
WT#2
T
C
Mito
T
C
Mito
25
20

Figure 4C

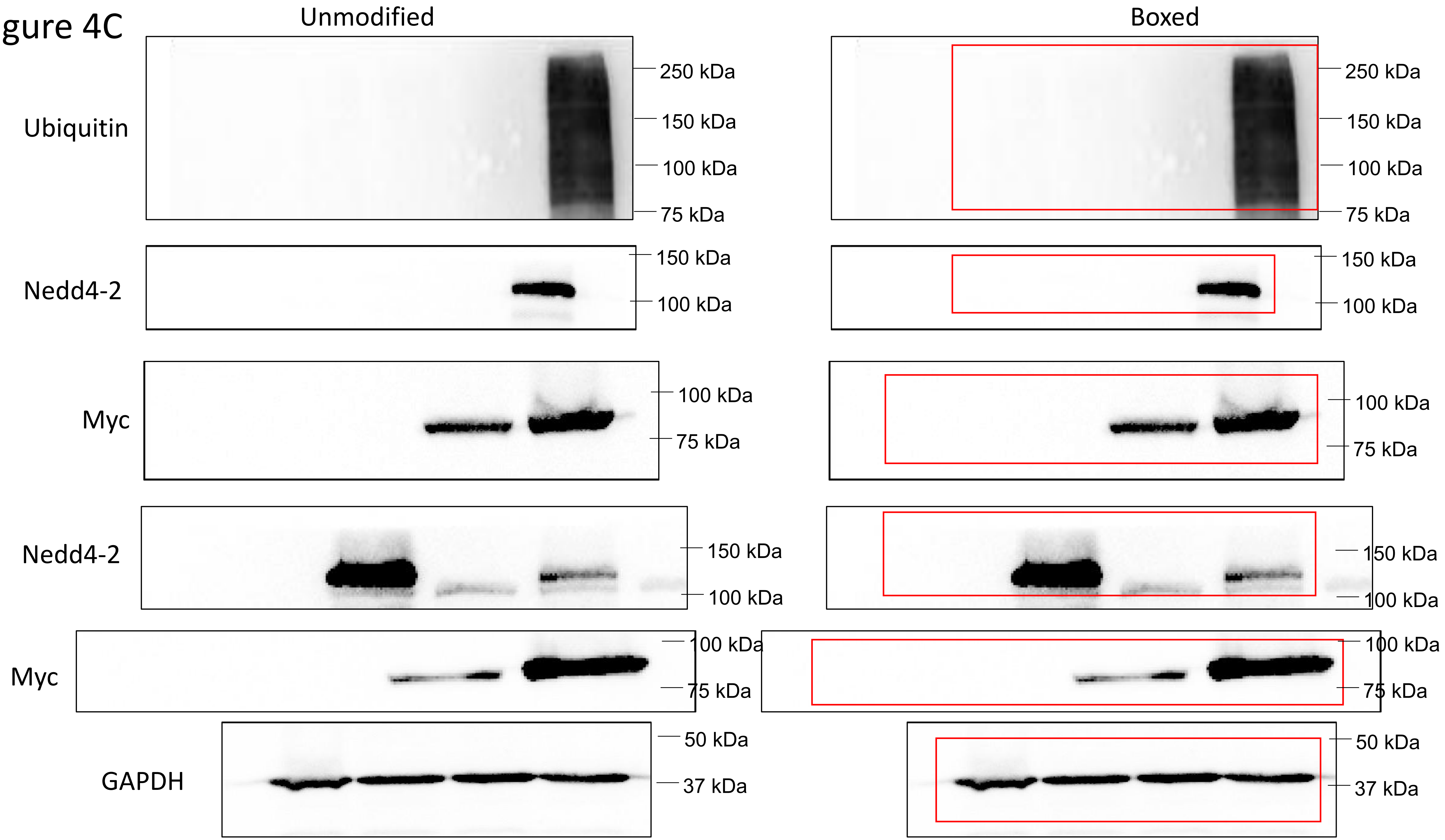
Unmodified
Boxed
Ubiquitin
250 kDa
150 kDa
100 kDa
75 kDa
Nedd4-2
150 kDa
100 kDa
Myc
100 kDa
75 kDa
Nedd4-2
150 kDa
100 kDa
Myc
100 kDa
75 kDa
GAPDH
50 kDa
37 kDa

Figure 4D_IP

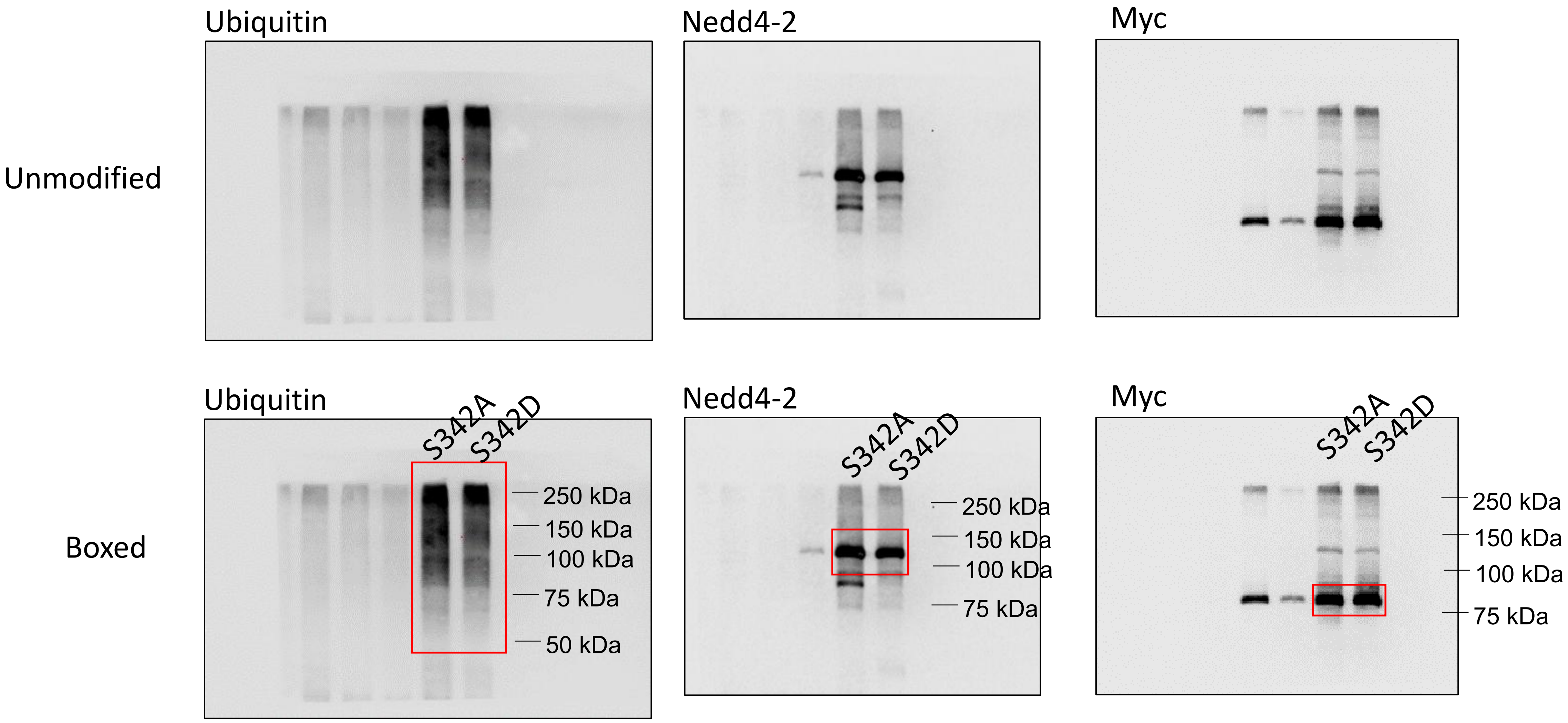
Ubiquitin
Nedd4-2
Myc
Unmodified
Ubiquitin
Nedd4-2
Myc
Boxed
S342A
S342D
250 kDa
150 kDa
100 kDa
75 kDa
50 kDa
S342A
S342D
250 kDa
150 kDa
100 kDa
75 kDa
S342A
S342D
250 kDa
150 kDa
100 kDa
75 kDa

Figure 4D_input

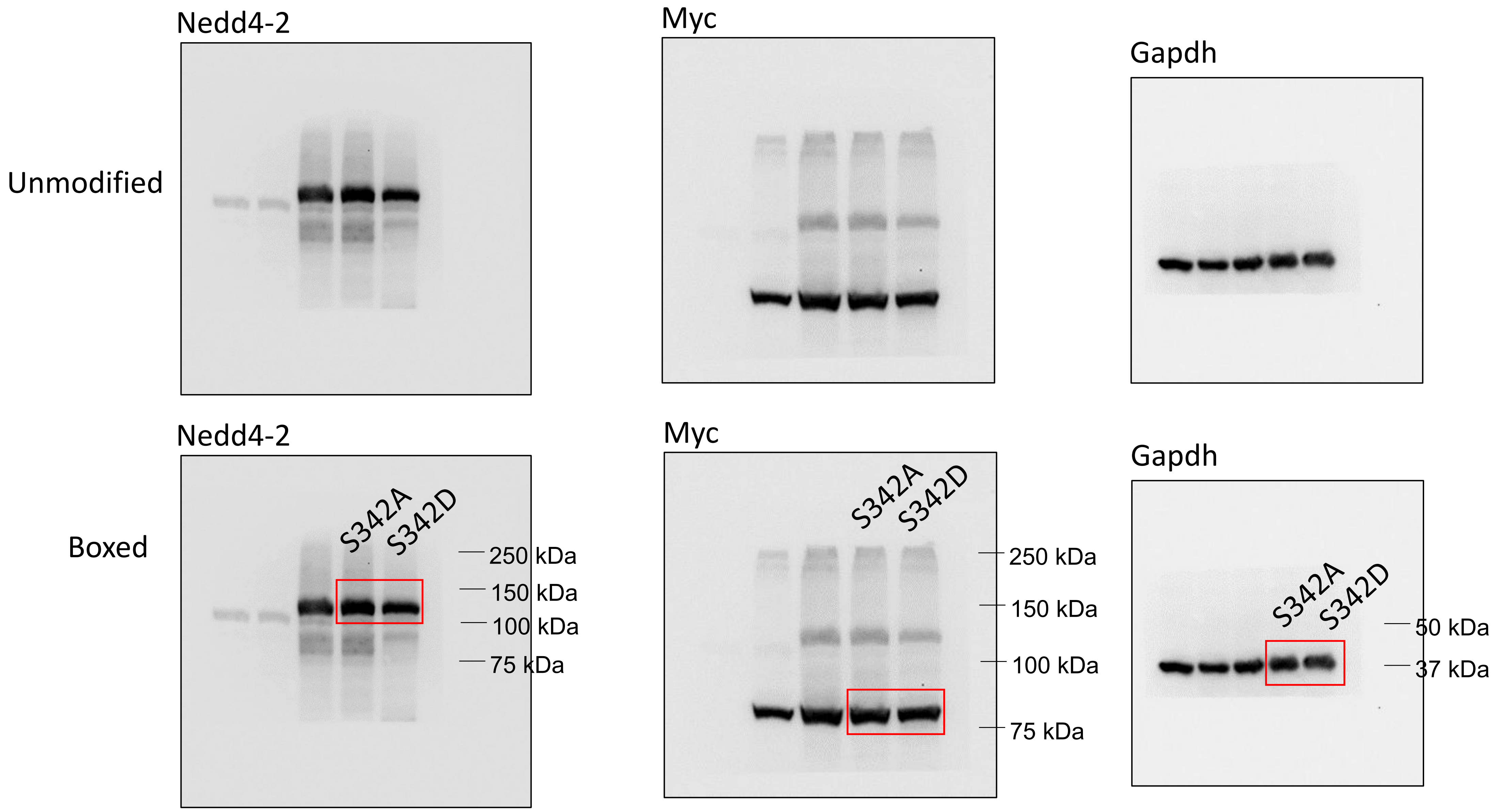

Figure 5A

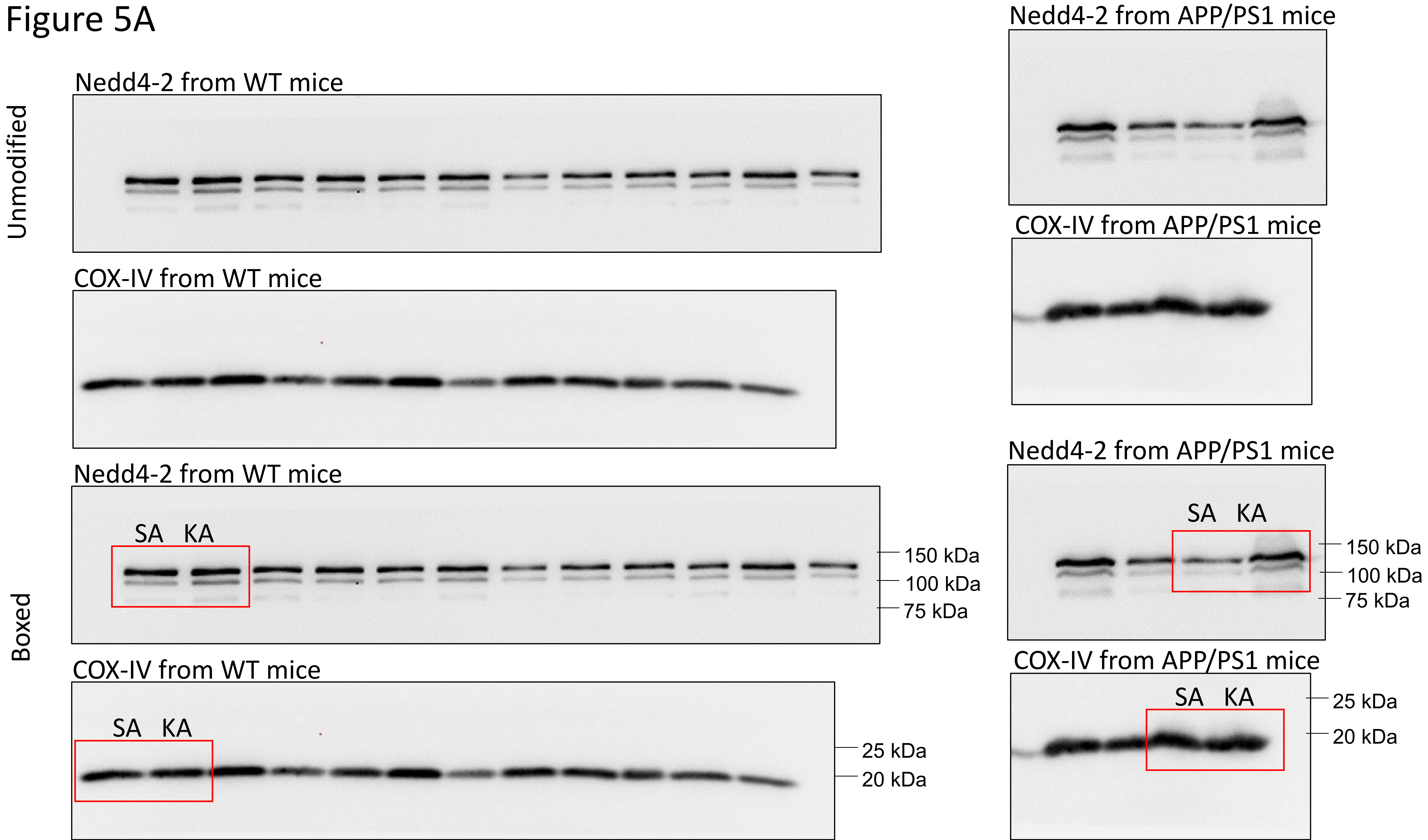

Unmodified
Nedd4-2 from WT mice
COX-IV from WT mice
Boxed
Nedd4-2 from WT mice
SA
KA
150 kDa
100 kDa
75 kDa
COX-IV from WT mice
SA
KA
25 kDa
20 kDa
Nedd4-2 from APP/PS1 mice
COX-IV from APP/PS1 mice
Nedd4-2 from APP/PS1 mice
SA
KA
150 kDa
100 kDa
75 kDa
COX-IV from APP/PS1 mice
SA
KA
25 kDa
20 kDa

Figure 5B

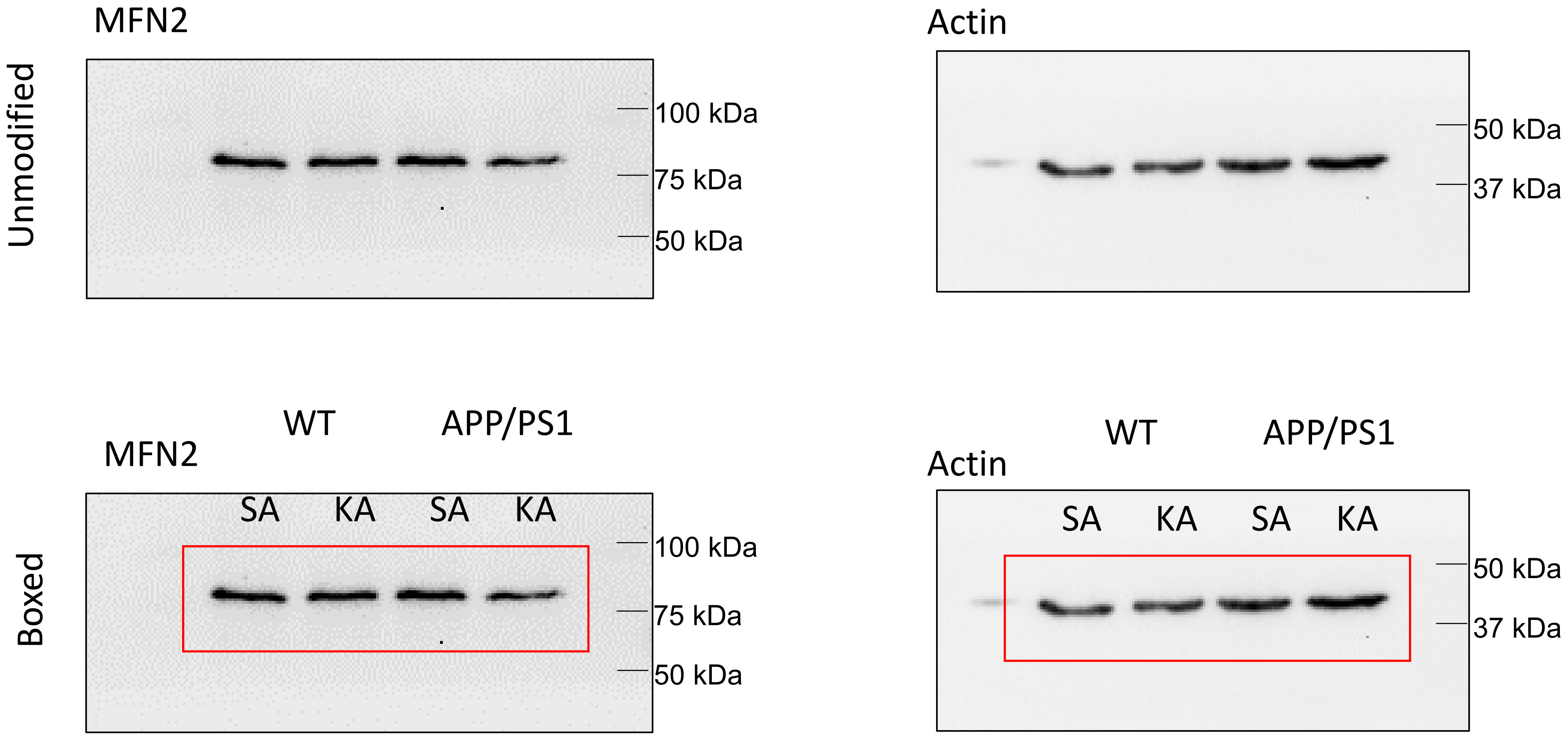

MFN2
Unmodified
100 kDa
75 kDa
50 kDa
Actin
50 kDa
37 kDa
MFN2
WT
APP/PS1
Boxed
SA
KA
SA
KA
100 kDa
75 kDa
50 kDa
Actin
WT
APP/PS1
SA
KA
SA
KA
50 kDa
37 kDa

Figure 5C_MFN2

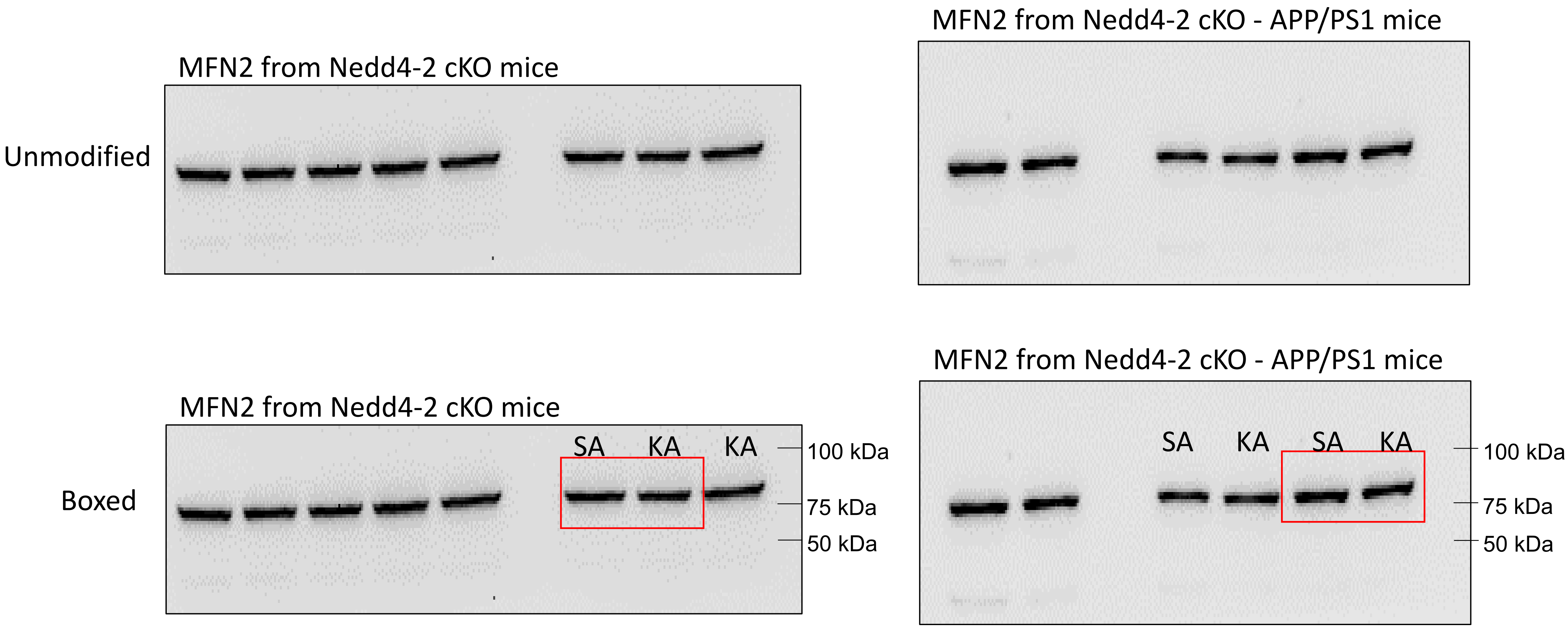

Figure 5C_Actin

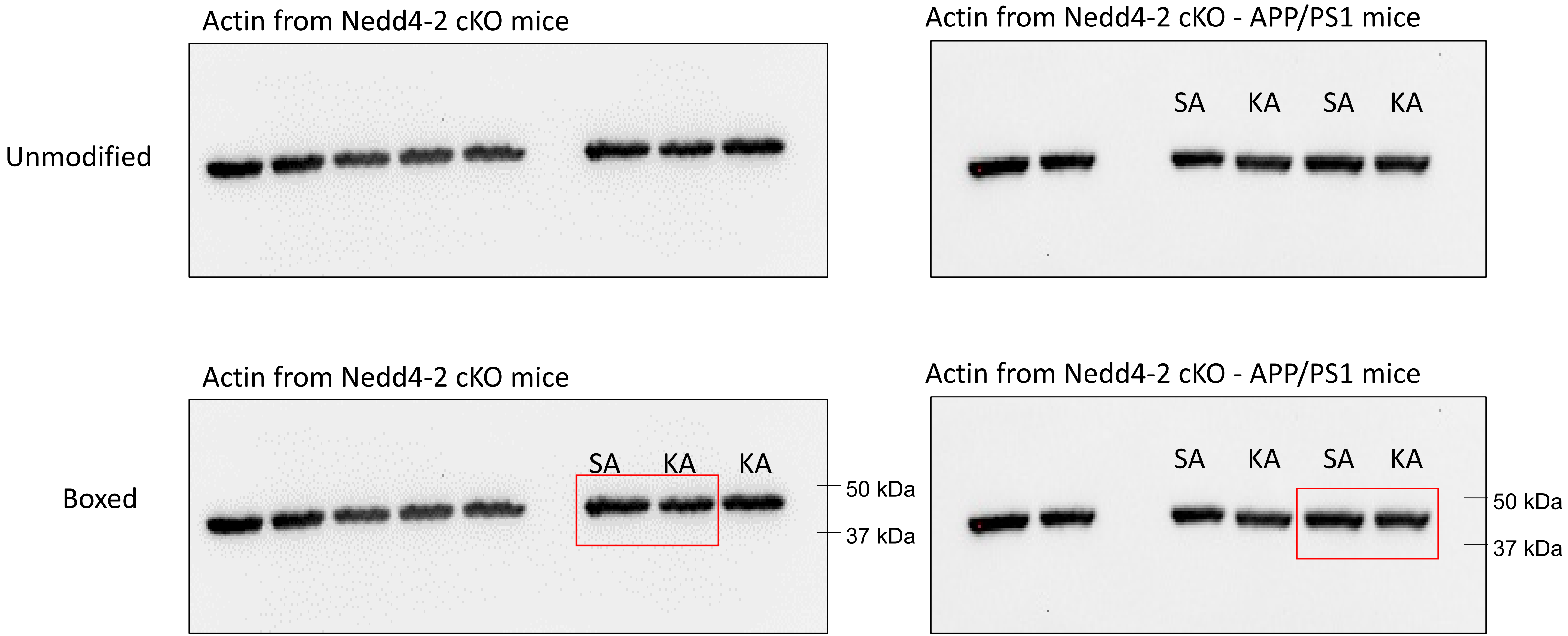

Supplemental Figure 1

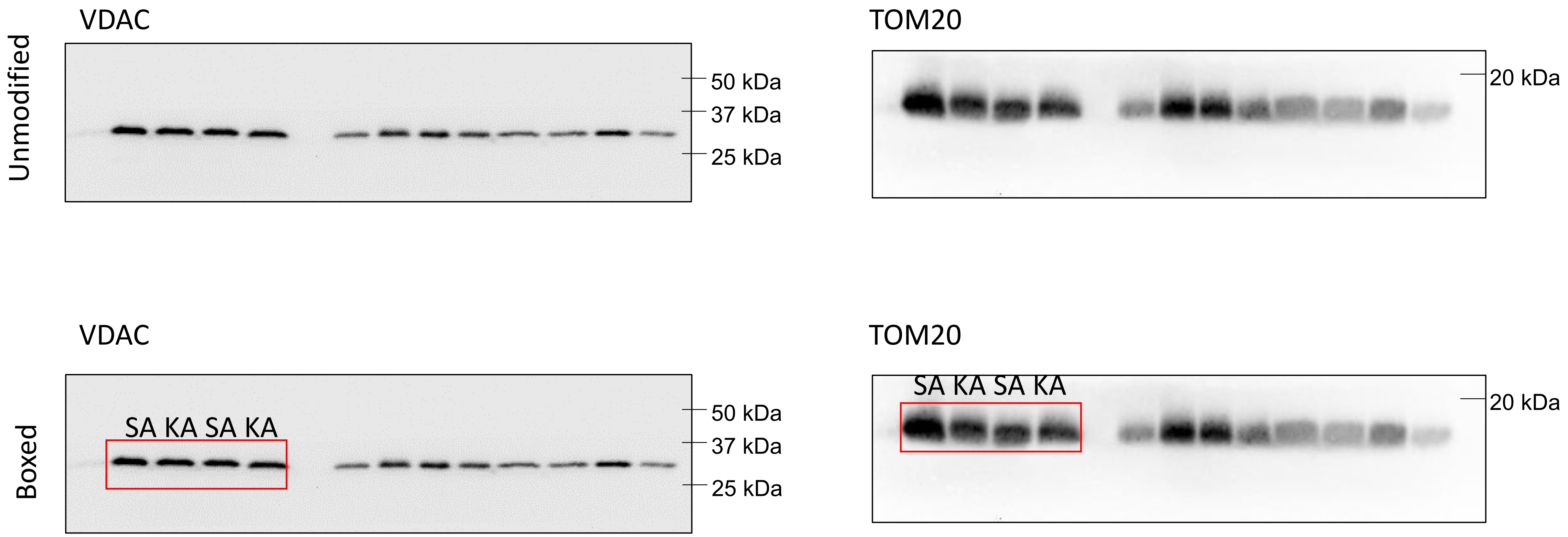

Supplemental Figure 1 - continued

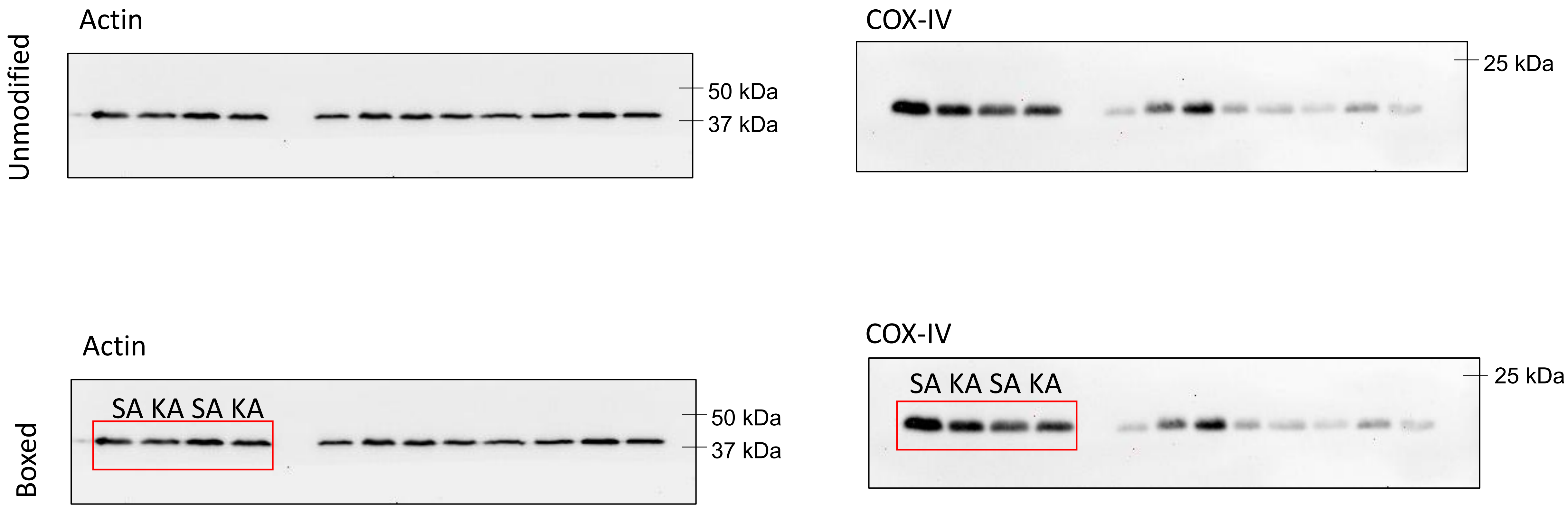

Supplemental Figure 3

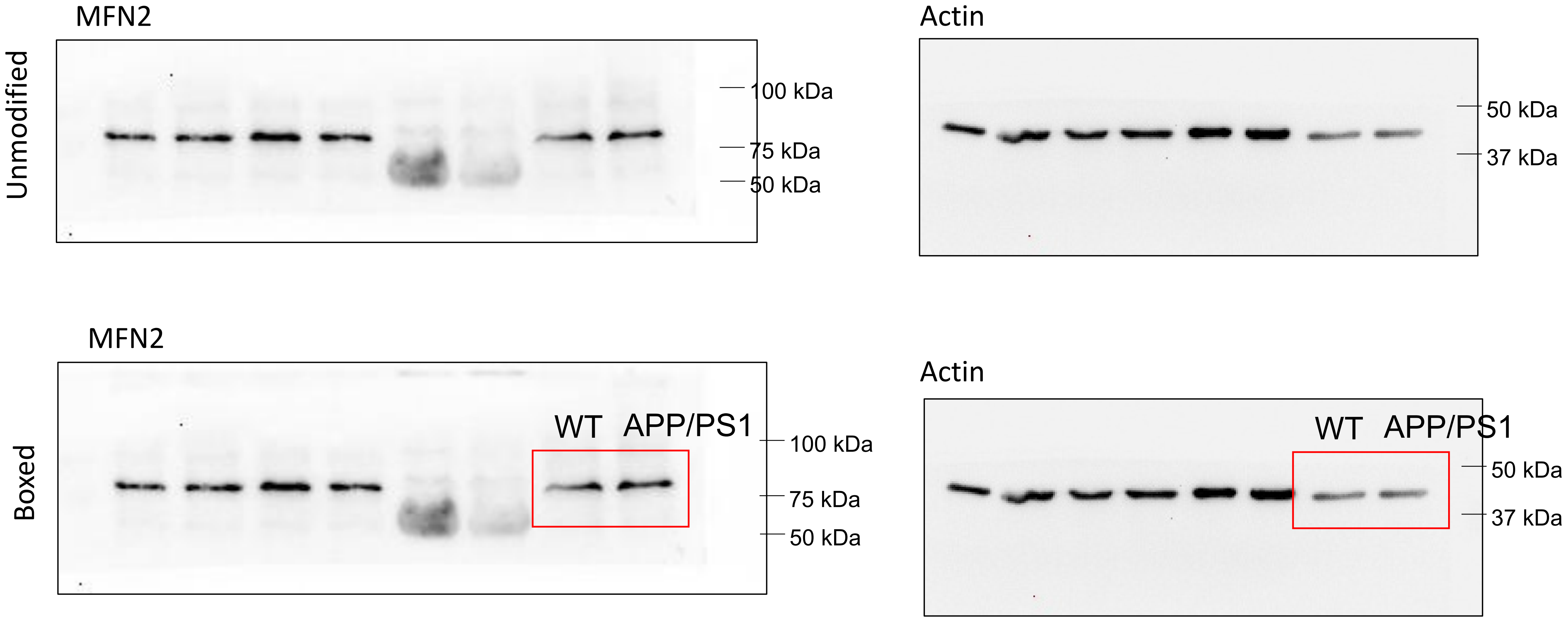

Supplement: Supplementary file 1 — Figure S1: Acute seizures do not alter the expression of other mitochondrial markers in WT or APP/PS1 mice. Representative western blots and quantification of Translocase of Outer Mitochondrial Membrane 20 (TOM20), Voltage‐Dependent Anion Channel (VDAC), mitochondria marker COX IV and β‐actin in purified mitochondria from 10‐week‐old wild‐type (WT) and APP/PS1 mice intraperitoneally injected with saline or kainic acid (15 mg/kg) for 2 h (n = 18–21 mice per group). Two‐way ANOVA with Tukey's test was used. Data are represented as mean ± SEM with ns: nonsignificant. Figure S2: An additional image showing partial colocalization between Nedd4 2 and COX IV. Immunocytochemistry images of Nedd4‐2 and mitochondrial marker cytochrome C oxidase subunit 4 (COX‐IV) in a cultured WT cortical neuron. An enlarged dendritic area was shown on the right. Scale bar: 10 μm. Figure S3: MFN2 is basally elevated in the total brain lysates of APP/PS1 mice at 10 weeks of age. Representative western blots of Mitofusin 2 (MFN2) and β‐actin in total brain lysates from 10‐week‐old WT and APP/PS1 mice (left) and quantification (right) (n = 12 mice per group). Student's t‐test was used. Data are represented as mean ± SEM with *p < 0.05. Table S1: Full Statistical Reports for all data in this study. Uncropped Full Images of Western Blotting Results. Table S2: Label‐free proteomics screening identifies proteins that are up‐ or down‐regulated in Nedd4‐2 cKO brains. [file JNC-170-e70440-s001.zip › jnc70440-sup-0001-FigureS1-S3@Figures S1-S3, Table S1, Raw Western blot images.pdf]
